# Deep immune B and plasma cell repertoire in non-small cell lung cancer

**DOI:** 10.3389/fimmu.2023.1198665

**Published:** 2023-06-15

**Authors:** Akshay J. Patel, Naeem Khan, Alex Richter, Babu Naidu, Mark T. Drayson, Gary W. Middleton

**Affiliations:** ^1^ Institute of Immunology and Immunotherapy (III), College of Medical and Dental Sciences, University of Birmingham, Birmingham, United Kingdom; ^2^ Institute of Inflammation and Ageing (IIA), College of Medical Sciences, University of Birmingham, Birmingham, United Kingdom

**Keywords:** non-small cell carcinoma, B cell, B lymphocytes, plasma cell, regulatory B cell, squamous cell carcinoma, adenocarcinoma

## Abstract

**Introduction:**

B cells, which have long been thought to be minor players in the development of anti-tumor responses, have been implicated as key players in lung cancer pathogenesis and response to checkpoint blockade in patients with lung cancer. Enrichment of late-stage plasma and memory cells in the tumor microenvironment has been shown in lung cancer, with the plasma cell repertoire existing on a functional spectrum with suppressive phenotypes correlating with outcome. B cell dynamics may be influenced by the inflammatory microenvironment observed in smokers and between LUAD and LUSC.

**Methods:**

Here, we show through high-dimensional deep phenotyping using mass cytometry (CyTOF), next generation RNA sequencing and multispectral immunofluorescence imaging (VECTRA Polaris) that key differences exist in the B cell repertoire between tumor and circulation in paired specimens from lung adenocarcinoma (LUAD) and squamous cell carcinoma (LUSC).

**Results:**

In addition to the current literature, this study provides insight into the in-depth description of the B cell contexture in Non-Small Cell Lung Cancer (NSCLC) with reference to broad clinico-pathological parameters based on our analysis of 56 patients. Our findings reinforce the phenomenon of B-cell trafficking from distant circulatory compartments into the tumour microenvironment (TME). The circulatory repertoire shows a predilection toward plasma and memory phenotypes in LUAD however no major differences exist between LUAD and LUSC at the level of the TME. B cell repertoire, amongst other factors, may be influenced by the inflammatory burden in the TME and circulation, that is, smokers and non-smokers. We have further clearly demonstrated that the plasma cell repertoire exists on a functional spectrum in lung cancer, and that the suppressive regulatory arm of this axis may play a significant role in determining postoperative outcomes as well as following checkpoint blockade. This will require further long-term functional correlation.

**Conclusion:**

B and Plasma cell repertoire is very diverse and heterogeneous across different tissue compartments in lung cancer. Smoking status associates with key differences in the immune milieu and the consequent inflammatory microenvironment is likely responsible for the functional and phenotypic spectrum we have seen in the plasma cell and B cell repertoire in this condition.

## Introduction

Lung cancer is the leading cause of cancer-related death worldwide (1). Surgery plays a crucial role in the diagnosis, staging, and definitive management of non-small cell lung cancer (NSCLC). Resection is the treatment of choice for stage I and II NSCLC and an important component of the multimodality approach for stage IIIA disease (2). The presence of occult micro-metastatic circulating tumor cells at the time of surgery, which cannot be detected by modern staging methods, is likely to drive recurrence after resection of the tumor bulk (3). Immune responses within the tumor microenvironment are increasingly implicated as determining factors of tumor progression and aggressiveness (4). Immune research in NSCLC has focused predominantly on T-cell immune biology, however the immune response is a complex interplay between the primary tumor and multiple immune cell types in the tumor microenvironment (5). The role of B cells in tumor survival has been extensively investigated in recent years, and although much is yet to be determined, there is clearly both a pro- and anti-tumor role in this disease (6). The presence of B cells has been shown to correlate with improved survival and lower relapse rates in ovarian, cervical, and NSCLC (7–9). Immunosuppressive B cells that produce IL-10 are linked to a tumour microenvironment that releases high levels of pro-inflammatory stimuli (10). B cell presence has been associated with improved responses to checkpoint blockade in various disease settings, along with the presence of tertiary lymphoid structures (11–13). More recently, plasma cells, which have long been thought to play a minor role in the development of anti-tumor responses, have been implicated as key players in the response to checkpoint blockade in lung cancer patients (14–16). The underlying mechanisms and in-depth role of plasma cells that make them central to clinical responses are still not well understood.

To explore the relationship between B and plasma cells and post-resection outcome in these early-stage cancers, we utilized deep phenotyping techniques with a B cell-specific CyTOF panel, robust high-dimensional display techniques, and regression models to analyze the importance of specific B cell immunophenotypes in the circulation and within the tumor microenvironment on various clinical correlates, including disease-specific outcomes. We present a mass cytometry-based atlas of the B-cell immune landscape in NSCLC patients using blood and tumor samples. This work expands considerably on our understanding of the immune cell milieu in this disease.

## Materials and methods

### Experimental model and subject details

Peripheral blood mononuclear cell (PBMC) layers from age-matched healthy donors were obtained from the Clinical Immunology Service at the University of Birmingham (UoB). Primary blood samples from advanced Non-Small Cell Lung Cancer (NSCLC) patients were obtained before surgical resection of the tumour in the outpatient clinic, tumour tissue was obtained fresh at the time of surgery. Written consent was provided under the UoB Research Ethics Approval, protocol 17/WM/0272. Tumour stage and histological subtype with molecular profiling was determined by a radiologist and pathologist respectively (**Supplementary Table 1**). The study cohort is described in **Supplementary Table 1**. Median follow-up in these patients is 3 years. Only one cancer exhibited an EGFR mutation (EGFR+ exon 19 deletion adenocarcinoma). Forty cancers (71%) were PDL1 negative.

We examined the risk factors as independent predictors of overall and disease-free survival on multivariate testing (**Supplementary Table 2**). Squamous cell carcinoma was associated with reduced risk of death (HR 0.17, p-0.014) and male gender was associated with an increased risk of death (HR 5.45, p=0.034). In terms of recurrence, advanced stage (III) was associated with an increased risk (HR 25.6, p=0.016) and adjuvant chemotherapy was associated with a reduced risk (HR 0.04, p=0.018).

### In-depth immunophenotyping of NSCLC samples using mass cytometry

We performed large scale mass cytometry analysis of paired NSCLC patient samples (peripheral blood samples and fresh matched tumour tissue from patients with IASLC stage I-III NSCLC (n=56) and 5 healthy age-matched donor samples (peripheral blood only). All patient peripheral blood samples were taken before surgical tumour resection. Cells were stained a B cell antibody panel (34 antibody markers) created for this study (**Supplementary Table 3**). The panel was designed to detect the expression of B cells at various stages of maturation (activated, transitional, marginal zone, follicular, germinal centre, class-switched and plasma) as well as rarer B cell populations such B regulatory (Breg) cells. The panel also included markers for natural killer cells, T cells and granulocytes.

### Sample preparation and acquisition

Peripheral blood mononuclear cells were harvested using BD vacutainer® CPT bottles (NH: ~130IU FICOLL™ 2.0ml). Following centrifugation, the cells were washed twice with RPMI 1640, and re-suspended in freezing media (sterilised mix of 90% heat inactivated foetal calf serum and 10% DMSO) at a density of 4-10 x10^6^/ml prior to cryostorage at -80°C.

### Tumour dissociation

Fresh lung resections samples were immediately taken to the histopathology suite where tissue from the tumour core and periphery was sampled to ensure as much TME representation as possible. One set of samples was immediately placed in sterile sealed container containing Miltenyi Tumour Storage Solution and stored in a fridge at 4°C [cell labelling and cytometric analysis specimen] and the second set of samples was immediately placed in a sterile sealed container containing formalin for fixation and stored in a fridge at 4°C [VECTRA immunofluorescence analysis specimen]. All samples were transported to the laboratory for processing and analysis within 24 hours.

For single cell analysis, tumour tissue was dissociated into a single cell suspension by combining mechanical dissociation with enzymatic degradation of the extracellular matrix, which maintains the structural integrity of the tissue. This was carried out according to the manufacturer protocol using the gentleMACS™ Octo dissociator (Miltenyi Biotech). All reagents were supplied by Miltenyi Biotech and reconstituted in a standardised way.

### RNA extraction

Tumour RNA was extracted and purified using the Qiagen RNeasy® Plus Mini Kit according to manufacturer protocol. Tumour samples were defrosted according to the protocol outlined above.

### Cell staining for mass cytometric acquisition

CyTOF antibody cocktails (cell surface and intracellular done separately) were prepared using pre-determined optimal titres and filtered using Ultrafree MC 0.1μm centrifugal filter units (Merck Millipore) to remove antibody aggregates. Cryopreserved cells were resuscitated for mass cytometry experiments by rapid thawing at 37°C, slow dilution with wash media and then centrifugation to pellet cells and remove freezing media. The cells were then filtered through a 35μm nylon mesh using 5ml tubes with cell strainer caps and then washed with MaxPar Cell Staining Buffer (CSB, Fluidigm). Cells were then incubated with 5 µl of Fc receptor blocking reagent (Human Trustain Fc blocking solution, Biolegend) for 10 min at room temperature and then immediately incubated with surface antibodies at room temperature for 30 min. During the last 2 minutes of this incubation, cells were incubated with 1 µM cisplatin to allow live cell (cisplatin-)/dead cell (cisplatin+) discrimination. The reaction was quenched with CSB (Fluidigm). Cells were then fixed and permeabilised for intracellular antibody staining using MaxPar Fix I Buffer (Fluidigm®) and MaxPar Perm-S Buffer (Fluidigm®) (2 washes) respectively. Stimulation of cells prior to intracellular antibody was not performed to avoid altering rare cellular phenotypes and investigate constitutive expression reflective of the microenvironment (17, 18). The cells were resuspended and 2 µl of Heparin solution (2kU/ml stock) was added to each sample to prevent non-specific binding of charged eosinophils for a total of 10 minutes. The intracellular antibody cocktail was then added to the cells. After gentle agitation, the suspension was left to incubate for 30 minutes at room temperature. Cells were then washed with buffer and resuspended in 500 µl of Cell Intercalation Solution (1:1000 Nucleic acid Rh^103^ Intercalator: Fix and Perm Buffer (Fluidigm®)) and incubated overnight at 4 °C.

### Preparation for data acquisition

The next day, samples were washed twice with cell staining buffer, re-suspended in 1 ml of MilliQ ddH2O, filtered through a 35-µm nylon mesh (5ml tubes with cell strainer caps, BD) and counted. Before analysis, samples were resuspended in MilliQ ddH2O supplemented with EQ four element calibration beads (Fluidigm®) at a concentration of 0.5-1.0 x 10^6^ cells/ml. Samples were acquired at 300 events per second on a Helios instrument (Fluidigm®) using the Helios 6.5.358 acquisition software (Fluidigm®). We collected a minimum range of 750,000 – 1.2 million cells per samples in order to maximise chances of detecting rarer B cell subsets. IL10 detection albeit low in the unstimulated mass cytometry cohort, was reflective of likely Breg populations given the surface phenotype. We performed corroborative work on a stimulated cohort of melanoma cells which showed no difference in IL10 when compared to the parallel unstimulated cohort (19). Individual.fcs files collected from each set of samples were concatenated using the. fcs concatenation tool from Fluidigm® (CyTOF normalisation software 2), and data were normalized based on EQ four element signal shift over time using the same tool.

### Antibody labelling and conjugation protocol

In-depth characterization of B cells within our cohort was performed using metal-tagged antibodies. Metal conjugated antibodies were purchased from Fluidigm or conjugated to unlabelled antibodies in-house. All unlabelled antibodies were purchased in carrier-free form and conjugated with the corresponding metal tag using the MaxPAR antibody conjugation kit (Fluidigm®) as per manufacturer’s instructions. Metal isotopes were acquired from Fluidigm. The concentration of each antibody was assessed after metal conjugation using a Nanodrop 2000 (ThermoFisher Scientific). Conjugated antibodies were diluted using PBS-based antibody stabilizer supplemented with 0.05% sodium azide (Sigma-Aldrich) to a final concentration of 200 µg/ml and were subsequently titrated to an optimal concentration for use. Provider, clone, and metal tag of each antibody used in this study are provided in **Supplementary Table 3**.

### Multiplex immunofluorescence analysis

The NSCLC samples were fixed in 4% isotonic formaldehyde for no more than 24 hours, dehydrated and embedded in paraffin. Sections (4-μm) were cut from each paraffin-embedded tissue and stained with hematoxylin and eosin (HE) to evaluate tumour pathology.

Formalin-fixed, paraffin-embedded (FFPE) tissue sections of (4μm) were baked for 2 h at 60°C before staining. Deparaffinization and antigen retrieval (pH9 for 20 minutes at 100°C) were performed on the Leica BondRx Automated IHC stainer. Primary antibody dilutions were optimized individually in a chromogenic DAB staining. Control tissue was stained with the Bond Polymer Refine Detection kit (DS9800) and evaluated by a pathologist for specificity. Each marker was then assessed by a single fluorescence staining, to optimise the fluorophores dilution and to generate a library for spectral separation, using the Opal Polaris 7 Colour Automation IHC Detection Kit (NEL871001KT) from Akoya Biosciences. Each marker was tested in the six different positions to evaluate the effect of the heat deactivation steps and the epitope stability and determine their sequence in the panel accordingly (**Supplementary Table 4**).

Slides were serially stained with the following antibodies: IL-10 (1:400), CD138 (RTU), anti-CD4 (1:200), -CD20 (1:200), -BCL6 (RTU) and CD8 (1:400), with an incubation of 30 minutes. Secondary antibody used was OPAL POLYMER HRP MS + RB (ARH1001EA) from Akoya Biosciences, incubated for 10 minutes. TSA-conjugated fluorophores used to visualize each biomarker were Opal 480, Opal 780, Opal 690, Opal 620, Opal 570 and Opal 520, with a 10-minute incubation. Opal 780 was incubated for 60 minutes, preceded by a 10-minute incubation in TSA-DIG. Slides were mounted with ProLong Diamond Antifade Mountant (Fisher Scientific Ltd, 15205739) and stored at 4°C before imaging. Image acquisitions (20 × magnification as multispectral images) were performed using the Vectra Polaris multispectral imaging platform (Akoya Biosciences), with the entire slide image being scanned and 7-10 representative regions of interest chosen by the pathologist. DAPI was used to count number of cells per slide. Negative controls (PBS instead of primary antibody) were run simultaneously with these samples.

### Quantification and data analysis

Files (.fcs) were processed and normalised as described and uploaded into Cytobank, populations of interest were manually gated, biaxial marker expression was performed for visualisation in Cytobank and events of interest were exported as. fcs files. CD19+ sample ‘clean-up’ was performed by gating on intact (103Rh+ DNA stain), no beads (140Ce−), live (194/195Pt−), no T-cells CD3− (141Pr), no immature granulocytes or natural killer cells CD16− (209Bi), CD45+ (89Y), and CD19+ B cells.

For the downstream analysis, the fcs files were loaded into the R (R Core Development Team, 2015). The signal intensities for each channel were arcsinh transformed with a cofactor of 5 (x_transf =asinh(x/5)). To facilitate differential discovery and analysis within our dataset, we employed a hybrid R-based pipeline largely based on the Bioconductor packages flowCore (20), FlowSOM (21), CATALYST (22), and diffCYT (23).

High-resolution, unsupervised clustering, and meta-clustering were performed using the FlowSOM and ConsensusClusterPlus packages, which allowed for scaling of millions of cells; therefore, no sub-sampling of the data was required (21, 22). Visualization of data was performed using the CATALYST package, which employs the ggplot2 R package as the graphical engine. To visualize high-dimensional cell populations in two dimensions, the Uniform Manifold Approximation and Projection (UMAP) algorithm (24) was applied to represent the characteristics of the annotated cell populations and identified biomarkers. Differential cell abundance analysis was performed using generalized linear mixed models (GLMM), and marker intensities using linear mixed models (LMM), implemented via the diffCYT package (21, 22), using a false discovery rate (FDR) adjustment (at 5% using the Benjamini-Hochberg method) for multiple hypothesis testing. To identify the main cell subsets using both B cell panels, FlowSOM was run with the parameter k ((x dim = 10 **x** ydim=10) = 100), defining the number of nearest neighbors, set to 100. The function then metacluster populations into two through maxk (default 20) clusters (21). To confirm and extend our biological discovery, the clustering algorithm was modified to detect a maximum of eight meta-clusters after assessing the initial unsupervised 20 meta-clusters for biological relevance, which was performed to deduce which clusters were deemed most important according to the algorithm. Furthermore, selective marker clustering algorithms were run to ensure true marker expression within clusters of interest. To further define specific B cell clusters, runs were carried out with Principal Component Analysis (PCA) pre-processing incorporating all markers on the panel (including those for T cell lineage) and then run without these markers (namely CD3, CD4, and CD8) to exclude those that are not expressed on B cells and likely to add “noise” in the cluster generation process and increase the impact of the biologically relevant markers (25, 26).

RNA library preparation was carried out using the Lexogen QuantSeq 3’ mRNA sequencing kit. FASTq files underwent quality control with Trimmomatic and Cutadapt R packages (27, 28). The high quality reads were then aligned to the genome in a process known as “mapping” using HISAT2 or STAR with subsequent quality control checks using RSeQC (29–31). The “counting” and generation of read count files were carried out with STAR (31), HTSeq or Subread packages (32, 33). The raw read count files were then imported into R for differential gene expression analysis with DESEq2 (34). Gene Set Enrichment analysis (GSEA), Gene ontology pathway analysis and KEGG pathway analysis were performed using the gage, clusterProfiler and pathview packages (35–37). Broadly speaking these analyses relied on ranking all genes in the data set, identifying the rank positions of all members of the gene set in the ranked data set and then calculating an enrichment score (ES) that represents the difference between the observed rankings and that which would be expected assuming a random rank distribution.

Statistical significance was determined using a 2-tailed non-parametric test for unpaired (Mann-Whitney U test) samples and the Kruskal-Wallis test for more than two independent groups. Univariate and Multivariate Stepwise Backward Elimination models were constructed. Overall and Disease-Free Survival were determined within the cohort, and inter-group differences were calculated using the log-rank method, which was carried out in R using the Survival and Survminer packages for Kaplan-Meier analysis and Cox proportional hazards regression, respectively. Suitable data cutpoints were determined using the pROC and cutpointr R packages for ROC and bootstrap analyses, respectively. Pairwise comparisons in longitudinal analyses were performed using the pairwise Wilcoxon rank sum test. Statistical significance was set to less than 0.05. Multiple comparison correction was applied using the Benjamini-Hochberg method.

### Data availability

Mass cytometry data: the data that support the findings of this study are available from the corresponding author upon reasonable request. This is largely owing to file size and logistics of patient confidentiality, reverse pseudonymisation and need for data to be kept at specific academic/research sites in line with the policies from individual trial protocols. Source data are provided with this paper. Correspondence and material requests should be directed to GWM.

### Code availability

The authors declare that the code for reproducibility of data are publicly available. Although the code was adapted from various sources, the underlying code itself was not modified or changed in any way and is readily available from the sources cited. The code can be made available from the corresponding author upon reasonable request.

## Results

### In-depth immunophenotyping reveals phenotypic diversity of circulating and intratumoural B cell populations

**Figure 1:**
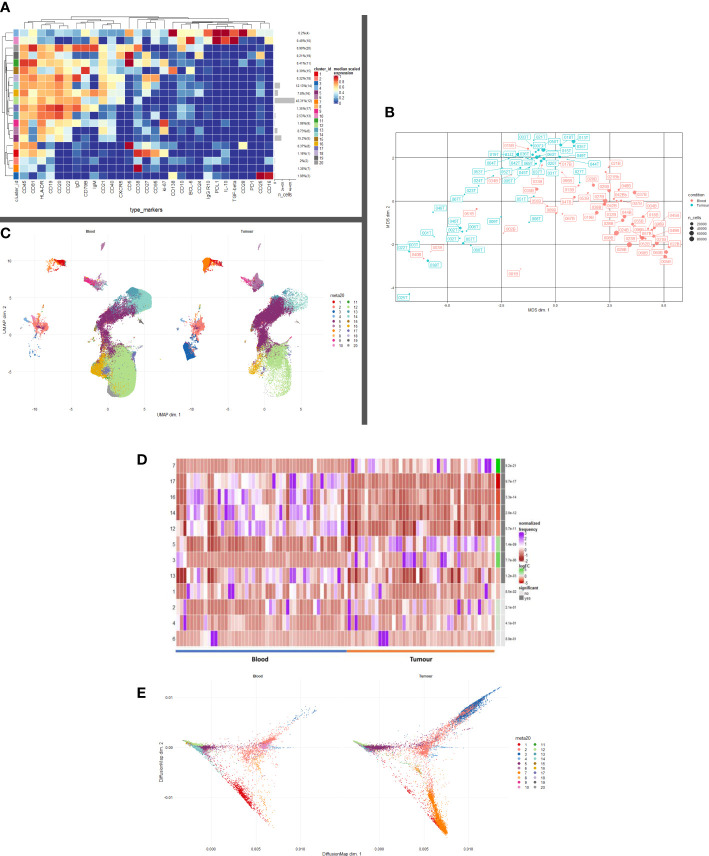
B and Plasma cell repertoire differences at the blood and tumour level. **(A)** A heatmap demonstrating the predominant 20 clusters in the B cell repertoire, as seen following FlowSOM clustering in the entire blood tumour population. Phenotyping markers are labelled along the x-axis. Clusters are labelled along the right y axis along with proportions as percentages of the overall population. Median scaled expression is shown in the intensity chart and used to determine expression of each marker. **(B)** Multi-dimensional scaling plot, Principal Component Analysis shows separation of CD19+ blood and tumour populations. Blood is illustrated in red and tumour in green as indicated by the colour chart in the right-hand column. **(C)** UMAP plots stratified according to condition, “blood” and “tumour”. All samples are randomly downsampled to account for equally representative populations across samples. Clusters are labelled in the right-hand chart. Clear differences exist between the two compartments (blood and tumour) as shown by the differential visual representation of each cluster. **(D)** Differential Abundance Heatmap illustrating 20 previously identified clusters (1A) (left hand column) with relative normalised abundance of each cluster by tissue compartment and individual patient (main panel). Tissue type is shown along the bottom x axis (B – blood, blue Line, T – tumour, orange line). The grey bars on the right-hand side indicate a p<0.05 accounting for multiple correction testing with Benjamini Hochberg. The log fold change is with respect to tumour. Patient to patient variability was treated as a random effect in order to improve the robustness of the model. A generalised linear mixed regression model was applied to determine significance of differential abundance between conditions (blood and tumour); the top eight clusters were of statistical significance as shown by the grey bars (7, 17, 16, 14, 12, 53 and 13). **(E)** Diffusion map stratified according to condition, “blood” and “tumour”. All samples are randomly downsampled to account for equally representative populations across samples.

CD19+ cells were taken forward into all further downstream analyses. In order to map cell phenotypes, FlowSOM clustering was performed, and expression of B cell clusters across different tissue compartments were visualised as a heatmap (**Figure 1A**) with heterogeneity in marker level displayed at single cell level using UMAP.

We defined 20 distinct B cell clusters at various stages of maturation (**Figure 1A**). We have enumerated all the clusters in **Table 1** (below) and described their likely phenotype based on surface marker expression and drawn conclusions from the original descriptions of these populations in the literature (38–50).

**Table 1:** B cell populations identified in blood and tumour compartments.

Cluster Number	Population
1	ki67^hi^ IL10+ CD27+ CD38+ Plasmablasts
2	Immature PDL1+ IL10+ CD138+ CD38+ Breg/Natural Regulatory Plasma cell
3	Ig- CD19^lo^ CD138^dim^ likely non-B cell phenotype, possibly Natural Killer T cell
4	Insignificant cluster (0.2%) cannot be identified
5	CD19^lo^ Ig- CD38- CD24^lo/-^ CD21^lo^ Memory
6	ki67^hi^ CD24 CD25 CD27 B10 Breg
7	CD19^lo^ CD38^hi^ CD24- CD27^lo^ IgD- Antibody Secreting Plasma cells
8	Immature PDL1+ IL10+ CD5^hi^ Breg
9	ki67^hi^ IL10+ CD27+ CD38+ Plasmablasts
10	Insignificant cluster (0.48%) cannot be identified
11	CD5^hi^ CD10+ CD27^hi^ CD38+ Transitional
12	Follicular (CD20^hi^ CD22^hi^ IgD+ IgM^lo^)
13	Double negative memory B cells (CD27^lo^ IgD- IgM-)
14	Fully affinity matured, class-switched B cells (CD27^hi^ IgD- IgM-)
15	Ig- CD138^hi^ CD19^lo^ (CD79B^hi^) Plasma cells*
16	Activated (IgM+ IgD+ CD25+ CD27+)
17	CD19+ IgD^hi^ IgM+ CD24- CD27- resting Naïve B cell
18	IgD+ IgM- atypical memory B cells
19	PD1+ CD5^hi^ CD25^hi^ IL10+ Breg
20	Transitional (IgM^hi^ IgD^hi^ CD24^hi^ CD38^hi^ CD10^hi^ CD5+)

The mostly frequently observed cluster was of the follicular B cell lineage (cluster 12, 43.41% of the total population (**Figure 1A**) characterised by high expression of CD20, CD22 and IgD and with lower levels of IgM. Activated (IgM+ IgD+ CD25+ CD27+) (cluster 16) and Transitional B cell (IgM^hi^ IgD^hi^ CD24^hi^ CD38^hi^ CD10^hi^ CD5+) clusters (cluster 20) comprised 7.8% and 0.99% of the total B cell population respectively. Antibody secreting plasma cells were characterised by low/dim CD19 expression and CD38 positivity (cluster 7, 1.38%). Cluster 15 comprised 0.39% of the total B cell population and was surface Ig- CD138^hi^ CD19^lo^ (CD79B^hi^), this may represent an atypical plasma cell population or an immature B cell population. Memory B cells were observed at various stages of maturation: Atypical IgD+ IgM- memory B cells (cluster 18, 0.32%), fully affinity matured, class-switched B cells (CD27^hi^ IgD- IgM-) (cluster 14, 12.13%) and double negative memory B cells (CD27^lo^ IgD- IgM-) (cluster 13, 2.53%).

Several B regulatory cell clusters were also identified including plasmablasts, PD-1+ CD5+ cells, PDL1+ cells and B10 populations (45–48). These were characterised using surface markers such as CD5, CD24, CD25, CD27, CD38, CD1d, TIM-1, PD1, PDL-1, TGF-β and intracellular cytokine expression of IL-10, were observed to varying frequencies (0.21%-2%). Clusters 1 and 9 are both likely to represent ki67^hi^ IL10+ CD27+ CD38+ plasmablasts. Clusters 2 and 8 represent immature PDL1+ IL10+ Bregs with cluster 8 also being CD5^hi^. Cluster 19 is a PD1+ CD5^hi^ CD25^hi^ IL10+ Breg. All B cells were unstimulated and thus IL-10 expression is representative of the *in vivo* immune milieu of NSCLC patients and healthy donors.

Unsupervised multi-dimensional scaling (principal component analysis) shows the broad differences in B cell repertoire between blood and tumour samples (**Figure 1B**). There is clear separation of these samples indicating differences in immunophenotype expression in the different environments. We performed comparative dimensionality reduction analyses (UMAP) between the paired blood and tumour samples from each patient (**Figure 1C**). On visual inspection, there are clear differences in cluster expression between the two environments. Broad enumeration of the two compartments identified a preponderance of early, maturating follicular and memory B cells in the blood, whereas more terminally differentiated plasma cells localised to the tumour microenvironment (TME).

*In blood*, there is visually higher expression of the following:

Cluster 1 – ki67+ CD27^hi^ CD38^hi^ CD95^hi^ IL10^int^ plasmablasts (Red)
Cluster 12 – CD20^hi^ CD21^hi^ CD22^hi^ Follicular (Light Green)
Cluster 13 – CD27^lo^ IgD- IgM- Double negative Memory (Teal)
Cluster 14 – CD27^hi^ IgD- IgM- Class-switched Memory (Aquamarine)
Cluster 16 – IgM+ IgD+ CD25+ CD27+ Activated (Mustard)
Cluster 17 – CD19+ IgD^hi^ IgM+ CD24- CD27- resting Naïve B cell (Lilac)
Cluster 19 – CD5^hi^ CD25^hi^ CD24+ PD1+ IL10^lo^ Breg (Dark Grey)
Cluster 20 – IgM^hi^ IgD^hi^ CD24^hi^ CD38^hi^ CD10^hi^ CD5+ Transitional/Breg spectrum (Light Grey)

*In tumour*, there is visually higher expression of the following.

Cluster 2 – Immature PDL1+ IL10+ CD138+ CD38+ Breg/Natural Regulatory Plasma (Peach)
Cluster 3 – Ig- CD19^lo^ CD138^dim^ likely non-B cell phenotype, possibly Natural Killer T cell (Royal Blue
Cluster 5 – CD19^lo^ Ig- CD38- CD24^lo/-^ CD21^lo^ Memory cells (Deep Purple)
Cluster 7 – CD19^lo^ CD38^hi^ CD24- CD27^lo^ IgD- Antibody Secreting Plasma cells (Orange)

Median marker expression analysis (**Supplementary Figure 1**) identified higher expression of early B cell and chemotactic surfaces markers in the circulation (CD5, CD20, CD21, CD27 and CXCR5), whereas expression of terminally differentiated and suppressive cells was noted in the TME (CD95, CD138, PDL1).

### Differential abundance analysis – blood versus tumour

We performed a differential abundance (DA) analysis of the defined cell populations (**Figure 1D**) reporting on all B cell clusters in the population. This method compares the proportions of cell types between the two clinical conditions and aims to highlight the populations that are present at significantly different ratios. In order to gain power to detect differences between conditions, we utilised a mixed model to model the response and patients were treated as a random effect thus formally accounting for patient to patient variability as described by Nowicka et al. (22, 51). DA analysis of the overall cell population identified eight clusters as significantly differentially abundant between the two environments (**Table 2**) below.

**Table 2:** Significant clusters on DA testing between blood and tumour compartments.

Cluster	Predominant Abundance	P value
3 - Ig- CD19^lo^ CD138^dim^ likely non-B cell phenotype, possibly Natural Killer T cell	Tumour	7.7*10^-6^
5 - CD19^lo^ Ig- CD38- CD24^lo/-^ CD21^lo^ Memory cells	Tumour	1.4*10^-9^
7 - CD19^lo^ CD38^hi^ CD24- CD27^lo^ IgD- Antibody Secreting Plasma cells CD19^lo^ CD38^hi^ CD24- CD27^lo^ IgD- Antibody Secreting Plasma cells	Tumour	9.2*10^-21^
12 - Follicular	Blood	5.7*10^-11^
13 - Double negative Memory	Blood	1.2*10^-3^
14 - Class-switched Memory	Blood	2.0*10^-12^
16 - Activated	Blood	3.3*10^-14^
17 - CD19+ IgD^hi^ IgM+ CD24- CD27- resting Naïve B cell	Blood	9.7*10^-17^

Lavin et al. demonstrated significantly different innate cell compartments in lung adenocarcinoma between healthy tissue and cancerous tissues (52). We used the publicly available dataset from this group and gated for CD19+ B cells and performed an unsupervised comparative principal component analysis between healthy lung tissue, blood, and tumour samples from stage I lung adenocarcinoma patients. The antibody panels used by this study were focused panels designed to interrogate specifically CD3+ T cell and NK cell compartments. The panels included few B cell specific markers other than CD19, CD27, CD38, PD1 and PDL-1 hence we were only able to discern broad subsets of B cells (52). The MDS plot (**Supplementary Figure 2**) illustrates separation between the three tissue types suggesting differences in the CD19+ B cell compartment between a) healthy and cancer tissue and b) blood and tumour of NSCLC patients. This supports the gross differences we observed in our dataset. We have ringed the broad populations in **Supplementary Figure 2** to better segregate the populations of interest. Owing to the significant heterogeneity between the antibody panel used by this group and ours, we were unable to drill deeper into the observed phenotypes. However, differential abundance analysis revealed several clusters which were significantly higher in the blood than in the TME.

### Plasma cell presence in the TME is on a phenotypic and functional spectrum

Clusters 2, 3, 5 and 7 are visually demonstrated as more abundant in the TME by the Diffusion Map (**Figure 1E**), the latter three being significantly more abundant. The bifurcating vectors of the diffusion map in **Figure 1E** represent two distinct axes of differentiation. Cluster 5 (deep purple) which starts at the early end of the maturation spectrum is a CD19^lo^ Ig- differentiating early plasma cell with potential to differentiate into an effector Ig producing plasma cell or a natural regulatory suppressive type of plasma cell. The top branch of diffusion map is made up of clusters 2 and 3 (peach and royal blue respectively) and these are phenotypically and functionally similar, expressing low levels of CD138, IL-10 and PDL1 indicating their suppressive nature. The bottom branch of the diffusion map is predominantly centred on cluster 7 (orange) which represents an effector Ig producing IL10- plasma cell. Thus, the TME is comprised of a terminally differentiated population of plasma cells which are being primed to an effector or suppressive phenotype.

**Figure 2:**
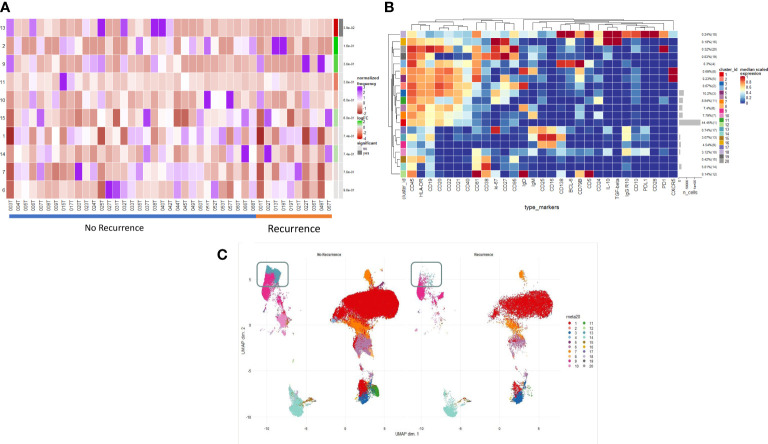
Intratumoural B and Plasma cell infiltration differs in early post-operative relapse. **(A)** INTRATUMOURAL (TME): Differential Abundance Heatmap illustrating 20 previously identified clusters in the TME (**B** heatmap) (left hand column) with relative normalised abundance of each cluster by recurrence and individual patient (main panel). Recurrence is shown along the bottom x axis (No recurrence - blue line, recurrence - orange line). The grey bars on the right-hand side indicate a p<0.05 accounting for multiple correction testing with Benjamini Hochberg. Patient to patient variability was treated as a random effect in order to improve the robustness of the model. A generalised linear mixed regression model was applied to determine significance of differential abundance between conditions (recurrence and no recurrence); there was only one cluster of statistical significance as shown by the grey bar (cluster 13, p=0.038). **(B)** A heatmap demonstrating the predominant 20 clusters in the B cell repertoire, as seen following FlowSOM clustering in tumour only. Phenotyping markers are labelled along the x-axis. Clusters are labelled along the right y axis along with proportions as percentages of the overall population. Median scaled expression is shown in the intensity chart and used to determine expression of each marker. **(C)** UMAP plots stratified according to condition, “recurrence” and “no recurrence” in the tumour. All samples are randomly downsampled to account for equally representative populations across samples. Clusters are labelled in the right-hand chart. Comparative UMAP demonstrating higher visual intra-tumoural expression of cluster 13 (CD138+, aquamarine cluster in the grey box) in non-recurrence patients.

**Figure 2A** illustrates the B cell populations within the TME. On deeper interrogation of these, we have identified several clusters (9–14) which exist on a phenotypic plasma cell spectrum. Cluster 14 (CD19- CD38^hi^ IgG+ early plasma cell) shows early features of plasma cell differentiation with CD19, high CD38 expression and early IgG expression. Cluster 9 (CD138+ CD25^hi^ IgG+ PDL1- IL10- terminally differentiated plasma cell) then shows CD138 expression with higher levels being expressed by cluster 13 which is a true effector terminally differentiated plasma cell population. Cluster 13 (CD138^hi^ IgG^hi^ PDL1^lo^ IL10- plasma cell) also shows early PDL1 expression which represents likely early transitioning to a regulatory plasma cell phenotype; cluster 10 (CD138^int^ IgG^int^ PDL1^int^ IL10^int^ plasma cell/regulatory suppressive) displays higher levels of PDL1 as well as producing IL10 becoming a suppressive plasma cell population. Cluster 11 (ki67^hi^ CD5+ CD10+ CD24^hi^ CD25+ CD27+ PD1+ transitional) represents a very early transitional cell population which has not yet become suppressive (IL10-, CD10+, PD1+). This focused assessment of the TME reinforces the phenotypic spectrum of infiltrating early/late plasma cells.

### Spatial analysis identifies localisation of immunosuppressive populations

Multiplexed immunofluorescent assays for CD4, CD8, CD20, CD138, IL-10 and BCL-6 were employed to visualise changes in immune infiltrate composition across NSCLC and with reference to those patients who developed post-operative recurrence. A significantly higher proportion of suppressive B cells (regulatory plasma CD138+ IL10+ and Breg CD20+ IL10+) infiltrate the tumour stroma as opposed to the tumour nest (p<0.0001) (**Supplementary Figures 4**, **5**). There were not any overall or compartmental differences in phenotype when stratified according to histology, stage, presence of lymphovascular invasion, presence of visceral pleural invasion or mortality.

### Structure of the immune landscape identifies phenotypes associated with effector function which in turn correlate with clinical outcome

We examined the differences in the B cell profile between those patients that recurred and those that did not. Within the TME, comparative testing revealed a single minimally significant subtle difference in B cell repertoire between recurrence and non-recurrence patients. Differential abundance testing revealed cluster 13 (**Figures 2A, B**) (CD138^hi^ IgG^hi^ PDL1^lo^ IL10- plasma cell) to be significantly more abundant in non-recurrence patients (**Figure 2C**, grey box) (p=0.03). There were no significant differences with respect to recurrence in the blood of these patients.

### Terminally differentiated effector and natural regulatory plasma cells are more abundant in ever smokers

**Figure 3:**
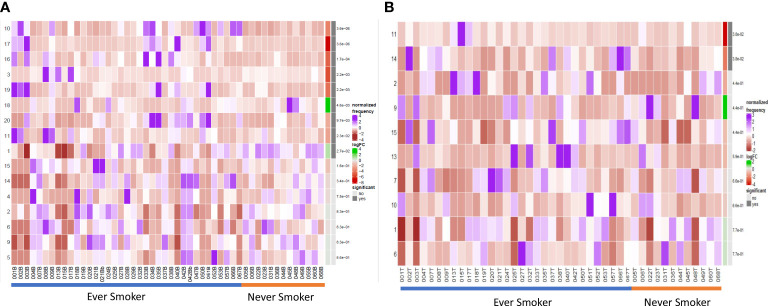
Differential B cell expression stratified to smoking status. **(A)** CIRCULATION (Blood): Differential Abundance Heatmap illustrating 20 previously identified clusters in the circulation (**Supplementary Figure 3** heatmap) (left hand column) with relative normalised abundance of each cluster by smoking status and individual patient (main panel). Smoking status is shown along the bottom x axis (Ever smokers - blue line, never smokers - orange line). The grey bars on the right-hand side indicate a p<0.05 accounting for multiple correction testing with Benjamini Hochberg. Patient to patient variability was treated as a random effect in order to improve the robustness of the model. A generalised linear mixed regression model was applied to determine significance of differential abundance between conditions (ever and never smokers); the top nine clusters were of statistical significance as shown by the grey bars (10, 17, 16, 3, 19, 18, 20, 11 and 1). **(B)** INTRATUMOURAL (TME): Differential Abundance Heatmap illustrating 20 previously identified clusters in the TME (**Figure 2B** heatmap) (left hand column) with relative normalised abundance of each cluster by smoking status and individual patient (main panel). Smoking status is shown along the bottom x axis (Ever smokers - blue line, never smokers - orange line). The grey bars on the right-hand side indicate a p<0.05 accounting for multiple correction testing with Benjamini Hochberg. Patient to patient variability was treated as a random effect in order to improve the robustness of the model. A generalised linear mixed regression model was applied to determine significance of differential abundance between conditions (ever and never smokers); the top two clusters were of statistical significance as shown by the grey bars (11 and 14).

We compared the B cell repertoire between never smokers and ever smokers according to the two different compartments using differential abundance analysis. Comparative cluster expression between the two groups identified a number of significantly differentially abundant clusters (**Figure 3A**, **Supplementary Figure 6**), which are summarised below (**Table 3**). Principal component analysis for blood (**Supplementary Figure 7**) shows a clear separation between the two groups. Median marker expression (**Supplementary Figure 8**) shows an increase of plasma cell markers, CD138 and IgG in ever smokers, as well as a higher level of IL-10 expression. Never smokers exhibit elevated expression of early-stage immature B cells, IgD, IgM and CD38 as well as homing marker CXCR5.

**Table 3:** Significant clusters on DA analysis according to smoking status.

Cluster (Blood)	Predominant Abundance	P value
1 – CD20+ CD22+ follicular	Never smokers	2.7*10^-2^
18 – CD5^hi^ CD25^hi^ CD24^int^ PD1+ transitional/Breg	Never smokers	4.6*10^-3^
3 – CD10+ CD38+ transitional	Ever smokers	2.2*10^-3^
10 – CD20 CD21^lo^ early	Ever smokers	3.6*10^-6^
11 – CD138^hi^ IgG+ ki67^int^ effector plasma	Ever smokers	2.2*10^-2^
16 – PDL1+ IL10+ CD27^hi^ IgD- IgM- class switched memory/regulatory)	Ever smokers	1.7*10^-4^
17 – CD138^lo^ PDL1^int^ IL-10^lo^ natural regulatory plasma cell	Ever smokers	3.6*10^-6^
20 – CD138^lo^ PDL1^int^ CD38^int^ IL-10- transitioning natural regulatory plasma cell	Ever smokers	9.7*10^-3^

Within the TME between the two groups, differential abundance analysis (**Figure 3B**) demonstrated that there was significantly higher infiltration of clusters 11 and 14 in ever smokers (p=0.038). These represent ki67^hi^ CD5+ CD10+ CD24^hi^ CD25+ CD27+ PD1+ transitional and CD19- CD38^hi^ IgG+ early plasma cell populations respectively (**Supplementary Figure 9**). Plasma cell infiltration appears higher in ever smokers in both the circulation and TME, but with clearly less marked phenotypic differences in the TME. CXCR5 expression is higher in never smokers.

### Next generation sequencing of TIBs in LUAD and LUSC tumour specimens

**Figure 4:**
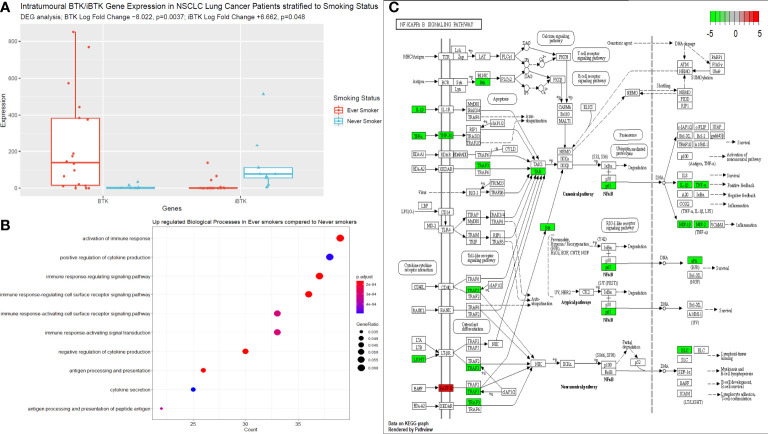
Next generating sequencing data stratified according to smoking status. **(A)** Box plot to illustrate the differences in BTK and iBTK expression between ever smokers and never smokers. Significance testing data is shown in the figure using differential gene expression analysis in R. **(B)** Gene set enrichment and ontology analysis plot demonstrating the key biological pathways that were significantly upregulated in ever smokers. P value is shown according to the colour chart on the right-hand side. **(C)** KEGG pathway analysis illustrated for Nf-ĸB signalling cascade. Genes highlighted in red indicate significantly upregulated genes in never smokers, this includes only BAFF. Genes highlighted in green, indicate significantly upregulated genes in ever smokers.

We performed bulk RNA sequencing of tumour specimens from both tumour tissues in 27 patients from our cohort. Following filtering, normalisation, and variance stabilising transformation of all genes sequenced in this dataset, 14739 genes were identified. Of these, 789 genes were significantly (p<0.05) differentially expressed between never smokers (n=9) and ever smokers (n=18); 99 had a log fold change (LFC) > 0 i.e., upregulated in the never smoker cohort and 690 had a LFC < 0 i.e., upregulated in the ever smoker cohort. Bruton Tyrosine Kinase (BTK) expression was significantly differentially expressed between groups, with higher expression in ever smokers (p=0.0037, following Benjamini-Hochberg correction, **Figure 4A**). Activation of immune responses, with cytokine production pathways and antigen processing and signalling pathways were significantly upregulated in ever smokers based on Gene Set Enrichment Analysis and Gene Ontology Analysis (**Figure 4B**). Critical pro-inflammatory genes and transcription factors in the Nf-ĸB signalling pathway were upregulated in ever smokers, whereas B-cell activating factor (BAFF) was identified as an upregulated gene in never smokers in this pathway (**Figure 4C**).

### Increased presence of terminally differentiated plasma and memory phenotypes in LUAD compared with LUSC patients

**Figure 5:**
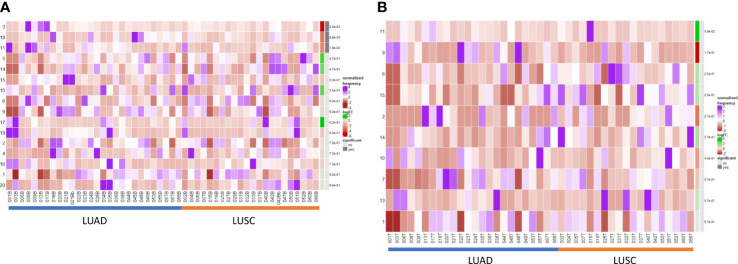
Differential B cell expression across histological subtypes. **(A)** CIRCULATION (Blood): Differential Abundance Heatmap illustrating 20 previously identified clusters in the circulation (**Supplementary Figure 3** heatmap) (left hand column) with relative normalised abundance of each cluster by histological subtype and individual patient (main panel). Tumour type is shown along the bottom x axis (Adenocarcinoma (LUAD) - blue line, Squamous cell carcinoma (LUSC) - orange line). The grey bars on the right-hand side indicate a p<0.05 accounting for multiple correction testing with Benjamini Hochberg. Patient to patient variability was treated as a random effect in order to improve the robustness of the model. A generalised linear mixed regression model was applied to determine significance of differential abundance between conditions (LUAD and LUSC); the top three clusters were of statistical significance as shown by the grey bars (3, 18 and 11). **(B)** INTRATUMOURAL (TME): Differential Abundance Heatmap illustrating 20 previously identified clusters in the TME (**Figure 2B** heatmap) (left hand column) with relative normalised abundance of each cluster by histological subtype and individual patient (main panel). Tumour type is shown along the bottom x axis (Adenocarcinoma (LUAD) - blue line, Squamous cell carcinoma (LUSC) - orange line). The grey bars on the right-hand side indicate a p<0.05 accounting for multiple correction testing with Benjamini Hochberg. Patient to patient variability was treated as a random effect in order to improve the robustness of the model. A generalised linear mixed regression model was applied to determine significance of differential abundance between conditions (LUAD and LUSC); none of the clusters were of statistical significance.

We performed a differential abundance analysis in the blood and TME compartments stratified by histological subtype (LUAD versus LUSC). In the circulation (**Figure 5A**), three populations were identified as significantly more abundant in LUAD and are demarcated by the blue boxes on the UMAPs in **Supplementary Figure 10**.

Cluster 3 – CD10+ CD38+ Transitional (royal blue) [p=0.0032]

Cluster 11 – CD138^hi^ IgG+ ki67^int^ Effector Plasma (green) [p=0.018]

Cluster 18 – CD5^hi^ CD25^hi^ CD24^int^ PD1+ Transitional/Breg (light purple) [p=0.0086]

Importantly, within the TME, none of the populations were significant between LUAD and LUSC on DA testing (**Figure 5B**) and thus B cell changes in the tumour are histotype agnostic.

## Discussion

Our study provides, to our knowledge, the most comprehensive immune cellular atlas of the B cell repertoire in NSCLC which focuses on the differences in B cell populations between the circulation and TME. We have demonstrated using high dimensional deep phenotyping that there are clear differences in the B cell repertoire between the circulation and intratumoural compartments. There is a preponderance of immature, naïve and follicular cells in the circulation with a higher level of infiltrating plasma cells in the TME. These plasma cells exist on a functional and phenotypic spectrum. We showed that in never smokers a higher proportion of immature B cells with high CXCR5 expression and ever smokers displayed higher degrees of plasma cell infiltration in both the TME and circulation. Functionally these cells exhibited a natural regulatory suppressive phenotype. We did not detect a significant B cell signature in different histological subtypes at the tumour level, however LUADs displayed higher levels of circulating terminally differentiated plasma cells and memory phenotypes. Lastly, when stratifying according to post-operative outcome, non-recurrence patients exhibited higher levels of infiltrating effector Ig+ IL-10- plasma cells which likely function to augment anti-cancer effector T cell responses, and directly mediate tumour cell death via antibody-dependent mechanisms.

Tumor-infiltrating B cells (TILBs) and plasma cells have been identified as important components of the TME and are linked to outcomes in lung cancer and responses to checkpoint blockade in advanced disease (11–13). Recently, a single cell analysis and spatial mapping of lung adenocarcinoma has shown for the first time highly enriched populations of plasma and memory B cells in tumor tissues with high levels of differentiation and somatic hypermutation (16). We significantly extend upon this study by profiling B cells in both blood and TME compartments in over 50 patients incorporating both LUAD and LUSC histological subtypes. We performed a direct comparison between blood and tumour and demonstrated a lack of surrogacy between the two compartments. Plasma cell infiltration was notably higher in the TME with more naïve resident B cells at the follicular and early memory stage residing within the circulation. Of note, we were able to detect an atypical IgD+ IgM- memory population in the blood, this has been described previously in the context of vaccine response and in circulation (49, 50, 53–56). Our TME data matched that of Hao et al. (16), however with the added granularity of the blood compartment comparison. Hao et al. showed high levels of CXCL13 production in the tumor tissue, which evolved with cancer progression, suggesting increased trafficking of these cells from tumor-derived signals into the TME (16). Our data demonstrated the circulating B cell populations were CXCR5+ which is analogous to the Hao data but further shows that the follicular and memory cells residing in the circulation, traffic into the TME dependent on the appropriate chemotactic/antigen-specific signal. Biologically, this is likely to represent a tumour driven polarisation of naïve B cells into terminally differentiated plasma cells which display an effector or suppressive phenotype. Multispectral Spatial analysis of the tumour tissue demonstrated a preponderance of suppressive cell types (CD20+ IL-10+ and CD138+ IL-10+) in the tumour stroma as opposed to the tumour nest. This localisation may be an immune evasion mechanism to dampen anti-tumour effector populations trafficking into the TME and to assist and facilitate the aforementioned functional polarisation of inbound immune cells.

Hao et al. (16) comprehensively assessed the TILB repertoire in tumor samples from 16 patients with LUAD using next-generation sequencing techniques. Late-stage memory and plasma cells with high levels of differentiation and somatic hypermutation, indicative of class-switching clones, were enriched in these early-stage tumors. We demonstrated similar infiltration of class-switched memory and effector plasma cells in the TME in LUAD, as well as a higher concentration of natural regulatory phenotypes compared to the LUSC TME. Patients with LUAD demonstrated elevated levels of effector plasma cells in the circulation compared to those with LUSC. A preponderance of earlier stage, less differentiated plasmablast/plasma cells was enriched in the LUSC TME. CIBERSORT analysis of LUAD (n=492) and LUSC (n=488) samples has shown similar plasma cell presence (9-10%); tumors lacking memory B cell infiltration exhibited a poor prognosis (57). A higher degree of immune heterogeneity has been postulated in LUSC, based on scRNA-seq data (58). The mutational burden and consequent neoantigen load in LUSC are often higher than those in LUAD (30), with the former exhibiting stronger smoking histories and p53 mutations. Trafficking of late-stage effector plasma cells into a more hostile inflammatory TME, such as that seen in LUSC, may explain the B cell dynamic differences between the two histological subtypes at the level of the circulation. This is a unique perspective offered by our analysis whereby comparative expression between histotypes has been demonstrated in the circulation but not in the TME.

Hao et al, showed in smokers, there is an increased prevalence of TILBs, in particular IgA+ and IgG+ plasma and memory cells (16). Elevated plasma cell infiltrates are correlated with better survival and response to immunotherapy. In parallel to plasma cell differentiation, memory cell infiltrate in these patients was more skewed towards a class-switched or germinal centre phenotype with lower degrees of BCR clonality in smokers and advanced-stage cancers (16).

We showed that later stage terminally differentiated effector plasma cells have been found in the circulation and TME of ever-smokers. Furthermore, we detected higher levels of suppressive natural regulatory plasma cells in the circulation of ever-smokers, which correlated with the elevated median expression of IL-10 in this group, presumably as a result of the greater inflammatory environment in smokers. This was supported by our functional RNA analysis of the tumour tissue from ever smokers, whereby these patients significantly over-expressed pro-inflammatory genes involved in Nf-ĸB signalling, in particular TNFα and IL-1β. Of note, Bruton’s Tyrosine Kinase (BTK) expression was significantly higher in ever smokers compared to never smokers and anti-correlated with inhibitor of BTK (iBTK) expression. BTK is a critical regulator of B cell development and has been investigated as a potential prognostic factor in LUAD with elevated levels corresponding to enriched immune cell activity and survival (59). Compelling murine data has shown BTK to be a critical regulator of matrix metalloproteinase-9 expression (MMP-9) in the alveoli and is a critical mediator of cigarette smoke induced inflammation in the lung parenchyma. ApoE^-/-^ mice exposed to cigarette showed less alveolar damage when concurrently treated with BTK inhibitors or had downstream siRNA induced silencing of MMP-9 activity (60). Targeting this molecule in autoinflammatory conditions may help to offset pulmonary hyperinflammation associated with cigarette smoking and reduce cellular damage burden potentially slowing the rate of carcinogenesis. B cell Activating Factor receptor (BAFF-R) was shown to be elevated in never smokers in our dataset. Murine data has shown that cigarette smoke may elevate BAFF expression by innate inflammatory immune cells with results lung inflammation (61–63). However, there is also evidence that cigarette smoke inhibits BAFF expression in the long-term in mice with resultant poor expression of mucosal IgA and hence augmented pulmonary inflammation and a reduced capability to cope with viral infection (64). There are likely various factors at play with may result in this paradoxical effect on BAFF/BAFF-R expression, in particular length of cigarette smoke exposure. Our cohort of never smokers, were completely naïve to cigarette smoke and may explain the uninhibited expression of BAFF-R. It is thus important to bear in the mind that other factors may well be at play in these patients that go beyond the smoking history, such as other genetic (including allied autoinflammatory conditions) and environmental factors (alcohol consumption, pollution index according to geographical location and other dietary and lifestyle factors).

Earlier stage B cells at the transitional and follicular stages were observed in never-smokers in the circulation, with a much higher median expression of CXCR5. Markedly elevated fractions of plasma cells have been observed in the TME of LUAD patients with a significant smoking history compared to never smokers; decreased B-cell clonality in smokers was also demonstrated (16). This also correlated with the degree of smoking history. This was particularly true for the fully differentiated plasma cell phenotype. Our data have demonstrated that there is a preponderance of late-stage effector plasma cells in ever-smokers, as shown by elevated CD138 and IgG expression in the circulation. As we have shown, the plasma cell differentiation axis is on a spectrum, with suppressive natural regulatory cells showing their presence in ever-smokers. Never smokers exhibited an elevated presence of early-stage B cells and transitional and follicular cells, with a preponderance of CXCR5^hi^ populations in the TME. The CXCL13-CXCR5 B cell chemokine axis is crucial for B cell recruitment and TLS formation. CXCR5+ B cells are highly enriched in early-stage LUADs (16). TCGA data showed progressive loss of CXCL13 expression with advancing pathological stages in LUAD. Exposure to cigarette smoke remodels the B cell repertoire not only in the TME but also in circulation. Exposure to tobacco smoke *in vitro* affects the evolution of the immune milieu in the LUAD TME (65, 66). Enrichment of differentiated memory B cell populations correlates with poor prognosis in tobacco-exposed LUAD (65). Significantly higher levels of class-switched memory B cells have been observed in the blood of current smokers (67). The number of point mutations in smokers with lung cancer is 10-fold higher than that in never smokers (34, 68, 69), and constant smoke-induced damage results in an evolving pattern of neoantigens in the lungs of these patients, shaping the adaptive immune response. We have shown elevated memory cells and differentiated B cells in ever-smokers, which presumably reflects the higher antigenic load in these patients. Indeed, high numbers of effector CD20+ cells in never smokers, where there is a lower mutational burden, correlates with favourable outcomes in LUAD (70). The lesser expression of “poised” CXCR5+ B cells in ever smokers, suggests that with increasing genomic perturbations and evolutionary dynamics in smokers, the ability to recruit effector subsets, activate anti-cancer responses and form TLS may well be lost as a result of enhanced tumor escape.

Increased intratumoural plasma cell infiltration has been reported to be associated with extended overall survival in NSCLC patients receiving anti-PDL1 treatment (14). Single-cell RNA sequencing data from the POPLAR trial (71) showed that the status of an immune module, determined by the high correlation found among activated T cells, IgG+ Plasma cells, and macrophages, termed lung cancer activated molecule (LCAM1), is associated with better progression-free survival in patients treated with anti-PDL1. The LCAM1^hi^ status showed a trend towards better overall survival in similarly treated patients (15). Murine models of castrate-resistant prostate cancer refractory to oxaliplatin treatment have demonstrated an increased presence of IgA+ Plasma cells that induce exhaustion of CD8+ T cells through PDL1 expression, as well as TGF-β and IL-10 production, a true regulatory suppressive plasma cell phenotype (72). Removal of this population enables the control of large tumors using oxaliplatin. This same suppressive population has been shown to accumulate in animal and human cases of inflammatory liver diseases, impeding anticancer effector T cell responses (73). Blockade of PDL1 in advanced-stage lung cancer could dampen the suppressive plasma cell phenotypes at play in the TME, thus shifting the balance towards the effector IgG-producing phenotypes.

We have demonstrated the infiltration of plasma cells into the TME across a phenotypic spectrum. Suppressive regulatory B cells can develop at any stage of B cell maturation, and the finding of CD138+ IL-10 producing plasmablasts provided evidence that this could occur even at the terminally differentiated end of the spectrum, with these cells demonstrating a BLIMP-1^LO^ phenotype and expressing switched IgG isotypes (47, 74). Plasma cell-derived cytokine-induced suppression has been shown to play a role in dampening inflammation within the CNS (75–79) and Recently, LAG-3+ IL-10+ CD138^hi^ plasma cells have been shown to rapidly induce IL-10 in a Toll-like receptor-driven manner following antigenic challenge, as well as being able to suppress IL-10 independently via PDL-1 and PDL-2, thus posing a favourable immunotherapeutic target (40, 80).

Our findings reinforce the phenomenon of B-cell trafficking from distant circulatory compartments into the TME. This differs across different histological subtypes and is influenced by the inflammatory burden in the TME, that is, smokers and non-smokers. In addition to the current literature, this study provides insight into the in-depth description of the B cell contexture in NSCLC with reference to broad clinico-pathological parameters which was previously limited to a small subset of adenocarcinoma patients in tumour specimens only.

It is important to bear in mind that the B cell milieu makes up 5% of the total PBMC fraction in the circulation of patients and the specific B cell phenotypes we are describing make up an even smaller fraction of these B and plasma cells. This is an inherent limitation of studying this area of immune biology and the significance we have observed between conditions has been assessed in an unsupervised and unbiaised manner treating patient to patient variability as a random effect to ensure reliability of data. Further larger scale cohort studies are required to confirm biological relevance however this study nonetheless adds an important, detailed comparative analysis to the lung cancer space with reference to B cell biology. The technique and analysis will be limited by manual gating methods, which are open to subjectivity, bias towards well-known subtypes, and inefficiency in larger datasets. The development of integrated machine learning methods will help bridge the gap with other OMICS data analyses and help infer developmental trajectories directly from cytometry data (81). Further Spatial mapping and functional studies are warranted to determine the exact pathogenic mechanisms underlying NSCLC. Nevertheless, this study provides increased granularity and definition of the plasma cell spectrum in lung cancer, and the dynamics of this axis in different histological and clinical disease settings.

## Data availability statement

The raw data supporting the conclusions of this article will be made available by the authors, without undue reservation.

## Ethics statement

The studies involving human participants were reviewed and approved by Written consent was provided under the UoB Research Ethics Approval, protocol 17/WM/0272. The patients/participants provided their written informed consent to participate in this study.

## Author contributions

AP and GM designed the experimental plan. AP carried out sample procurement, processing, data collection and analysis. AP and GM interpreted the results and constructed and designed the manuscript. NK, AR, BN and MD provided constructive feedback to the design and layout of the manuscript. All authors contributed to the article and approved the submitted version.

## Supplementary material

Supplementary Figure 1 Scaled median marker expression box plots illustrates phenotyping marker expression stratified to blood and tumour compartments across all cells in the population. Blood is illustrated in red and tumour in green as indicated by the colour chart in the right-hand column.

Supplementary Figure 2 Multi-dimensional scaling plot, Principal Component Analysis shows separation of CD19+ blood, tumour and normal lung tissue based on public data from Lavin et al. (52).

Supplementary Table 1 An overview of the demographic data of the cohort. This includes histopathological data as well as outcome data (mortality, recurrence rates and overall/disease-free survival).

Supplementary Table 2 Multivariate cox proportional hazards modelling illustrating the significant independent predictors of overall and disease-free survival in our cohort.

Supplementary Table 3 Antibody B cell marker panel for mass cytometry.

Supplementary Table 4 Markers of interest for multiplex immunofluorescence imaging with VECTRA.
