# Supplementary figures and images for: Deep immune B and plasma cell repertoire in non-small cell lung cancer
Source: Front Immunol. 2023 Jun 15;14:1198665. doi: 10.3389/fimmu.2023.1198665 (PMC10311499; doi:10.3389/fimmu.2023.1198665)

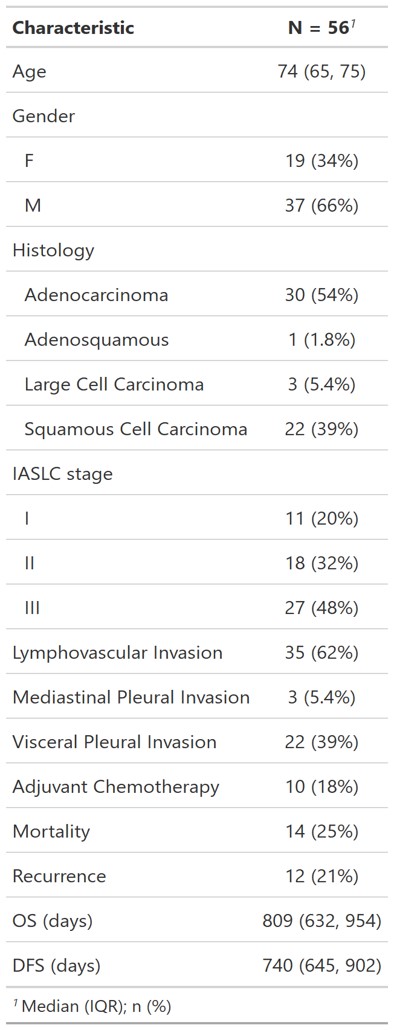

Supplement: Supplementary Figure 1 — Scaled median marker expression box plots illustrates phenotyping marker expression stratified to blood and tumour compartments across all cells in the population. Blood is illustrated in red and tumour in green as indicated by the colour chart in the right-hand column. [file Image_1.jpeg]

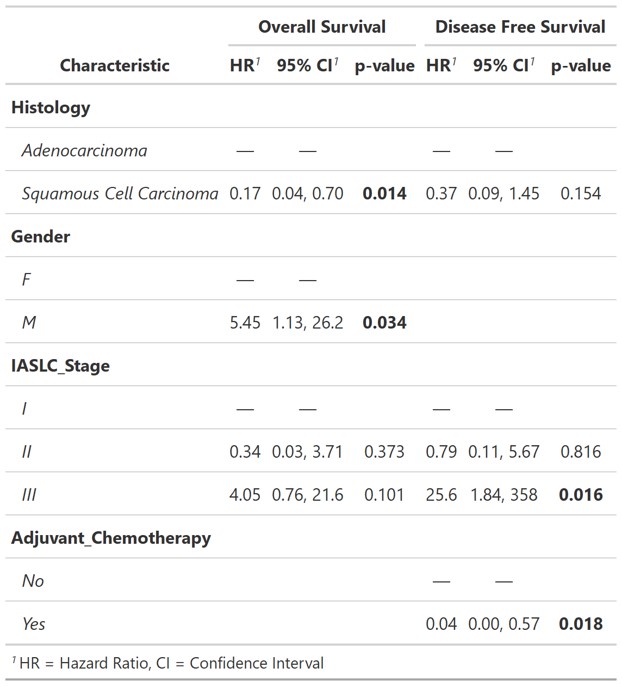

Supplement: Supplementary Figure 2 — Multi-dimensional scaling plot, Principal Component Analysis shows separation of CD19+ blood, tumour and normal lung tissue based on public data from Lavin et al. (52). [file Image_2.jpeg]

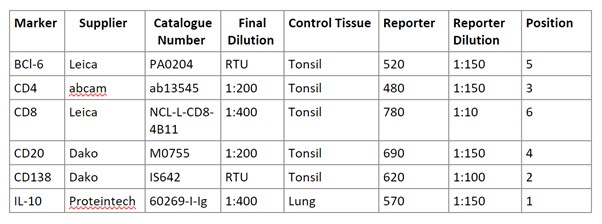

Supplement: Supplementary file 3 [file Image_3.jpeg]
