# Supplementary material for: Deep immune B and plasma cell repertoire in non-small cell lung cancer
Source: Front Immunol. 2023 Jun 15;14:1198665. doi: 10.3389/fimmu.2023.1198665 (PMC10311499; doi:10.3389/fimmu.2023.1198665)
Supplement: Supplementary Table 1 — An overview of the demographic data of the cohort. This includes histopathological data as well as outcome data (mortality, recurrence rates and overall/disease-free survival). [file Table_1.docx]

Supplementary Table 3. Antigen markers with corresponding metal tags for B cell mass cytometry panel

| **Metal Tag** | **Antibody** | **Clone** | **Source** |
| --- | --- | --- | --- |
| 89Y | CD45 | HI30 | Fluidigm |
| 141Pr | CD3 | UCHT1 | Fluidigm |
| 142Nd | CD19 | HIB19 | Fluidigm |
| 144Nd | CD38 | HIT2 | Fluidigm |
| 145Nd | CD81 | 5A6 | Fluidigm |
| 146Nd | IgD | IA6-2 | Fluidigm |
| 147Sm | CD20 | 2H7 | Fluidigm |
| 148Nd | CD8a | SK1 (Custom) | Biolegend |
| 149Sm | CD25 (IL2R) | 2A3 | Fluidigm |
| 150Nd | CD138 | DL-101 | Fluidigm |
| 151Eu | HLA-DR | G46-6 | Fluidigm |
| 152Sm | CD21 | BL13 | Fluidigm |
| 153Eu | Ig Lambda | MHL-38 (Custom) | Biolegend |
| 154Sm | IgG | R10 (Custom) | Biolegend |
| 155Gd | CD279 (PD-1) | EH12.2H7 | Fluidigm |
| 156Gd | CD274 (PD-L1) | 29E.2A3 | Fluidigm |
| 158Gd | CD10 | HI10a | Fluidigm |
| 159Tb | CD22 | HIB22 | Fluidigm |
| 160Gd | Ig Kappa | MHK-49 | Fluidigm |
| 161Dy | CD5 | UCHT2 (Custom) | Biolegend |
| 162Dy | CD79B | CB3-1 | Fluidigm |
| 163Dy | BCL-6 | K112-91 | Fluidigm |
| 164Dy | CD95/Fas | DX2 | Fluidigm |
| 165Ho | CD40 | 5C3 | Fluidigm |
| 166Er | IL-10 | JES3-9D7 | Fluidigm |
| 167Er | CD27 | L128 | Fluidigm |
| 168Er | Ki-67 | B56 | Fluidigm |
| 169Tm | CD24 | ML5 | Fluidigm |
| 170Er | TGF-Beta | TW4-6H10 (Custom) | Biolegend |
| 171Yb | CD185 (CXCR5) | 51505 | Fluidigm |
| 172Yb | IgM | MHM-88 | Fluidigm |
| 175Lu | CD28 | CD28.2 (Custom) | Biolegend |
| 176Yb | CD4 | RPA-T4 | Fluidigm |
| 209Bi | CD16 | 3G8 | Fluidigm |
