# Supplementary material for: Deep immune B and plasma cell repertoire in non-small cell lung cancer
Source: Front Immunol. 2023 Jun 15;14:1198665. doi: 10.3389/fimmu.2023.1198665 (PMC10311499; doi:10.3389/fimmu.2023.1198665)
Supplement: Supplementary file 5 [file Presentation_1.pptx]

## Slide 1
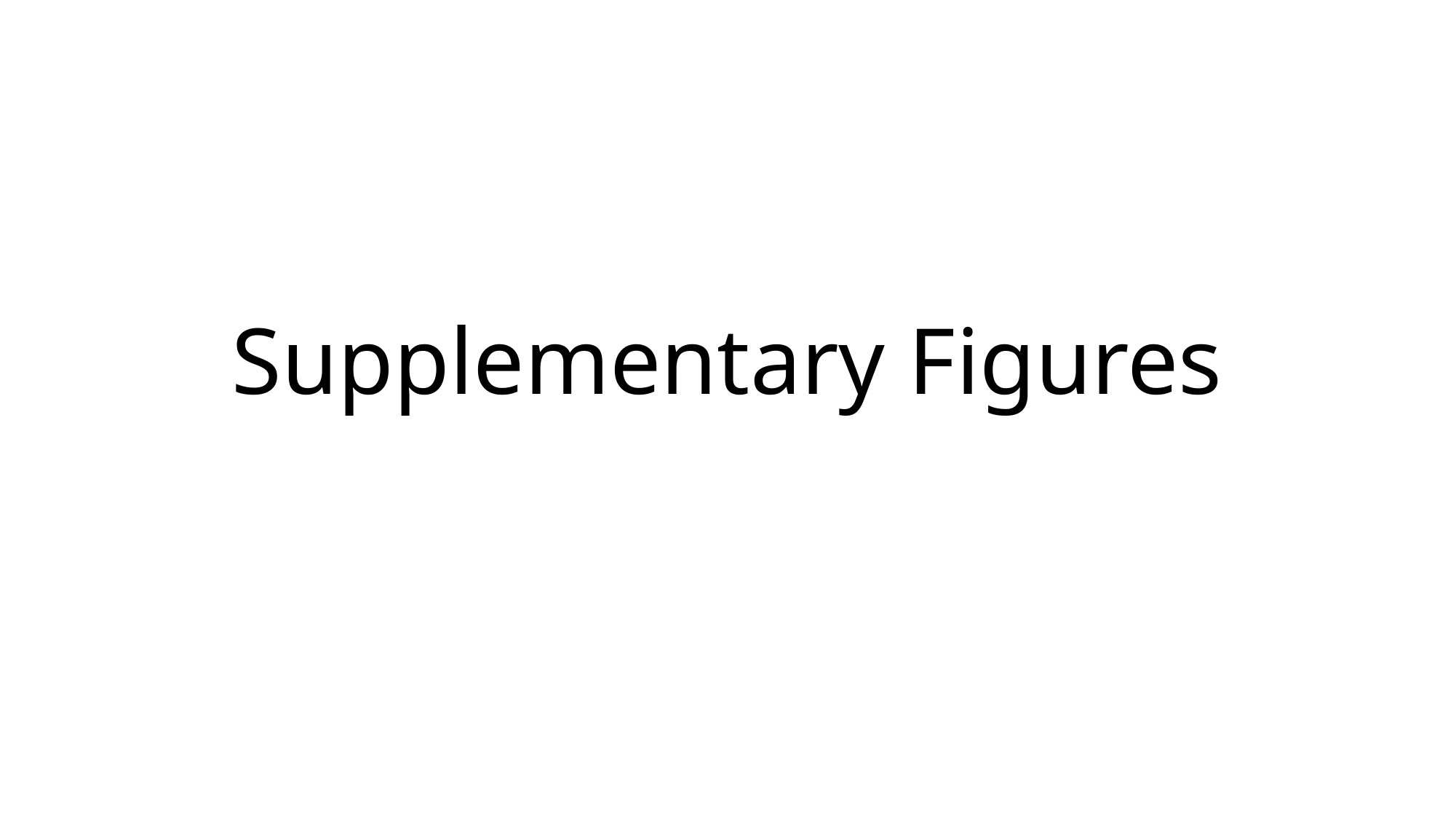

# Supplementary Figures

## Slide 2
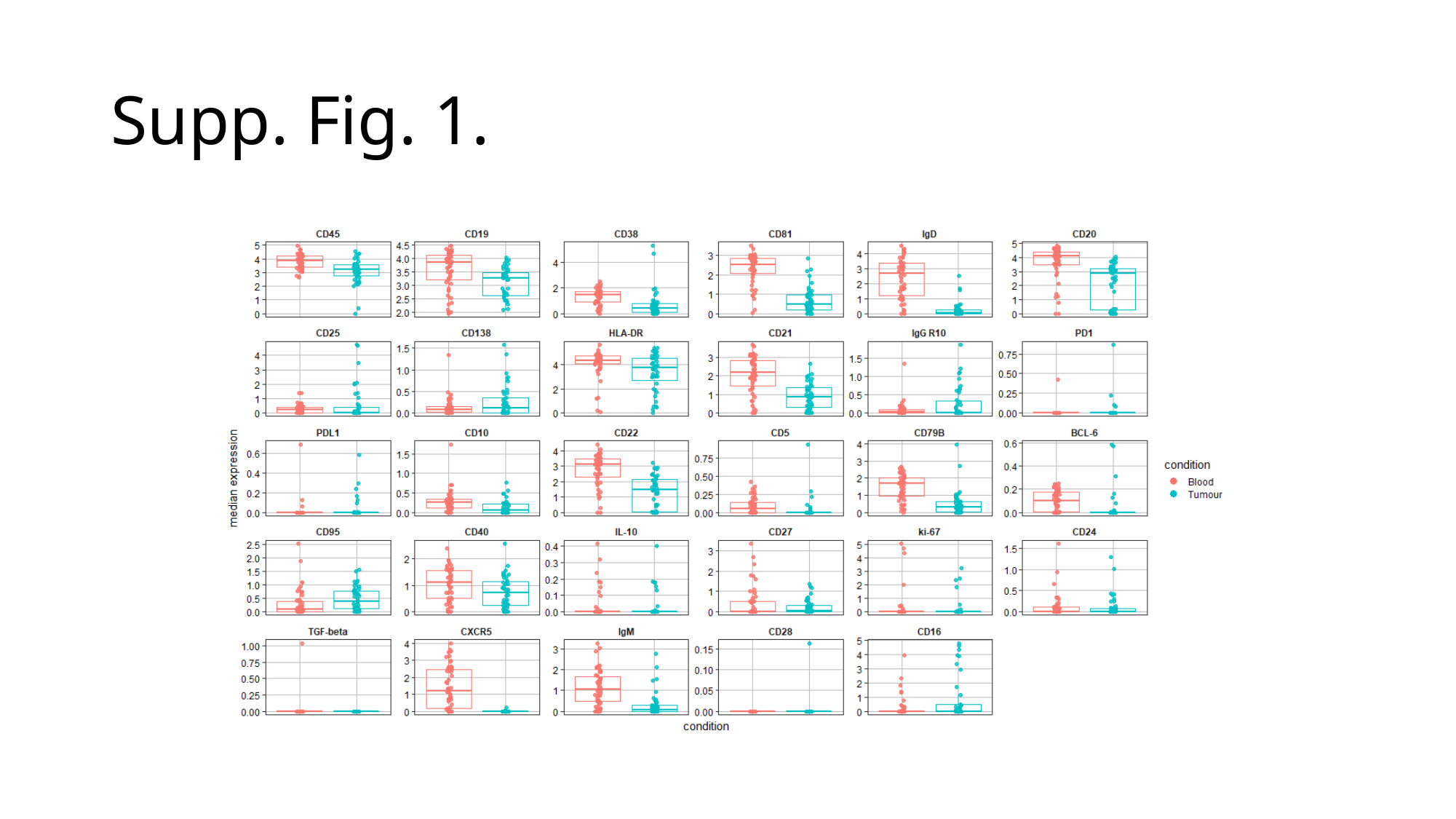

# Supp. Fig. 1.

## Slide 3
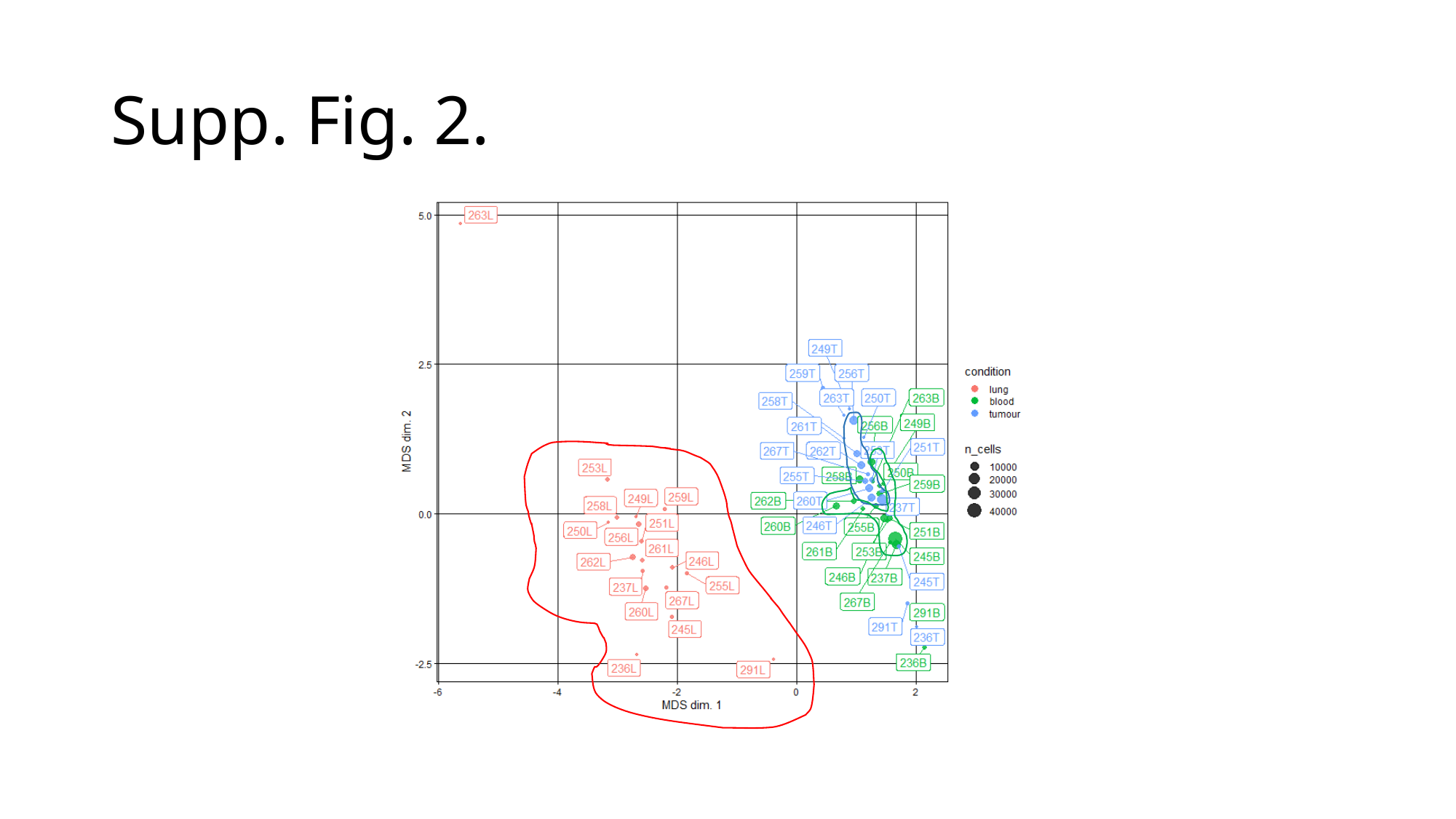

# Supp. Fig. 2.

## Slide 4
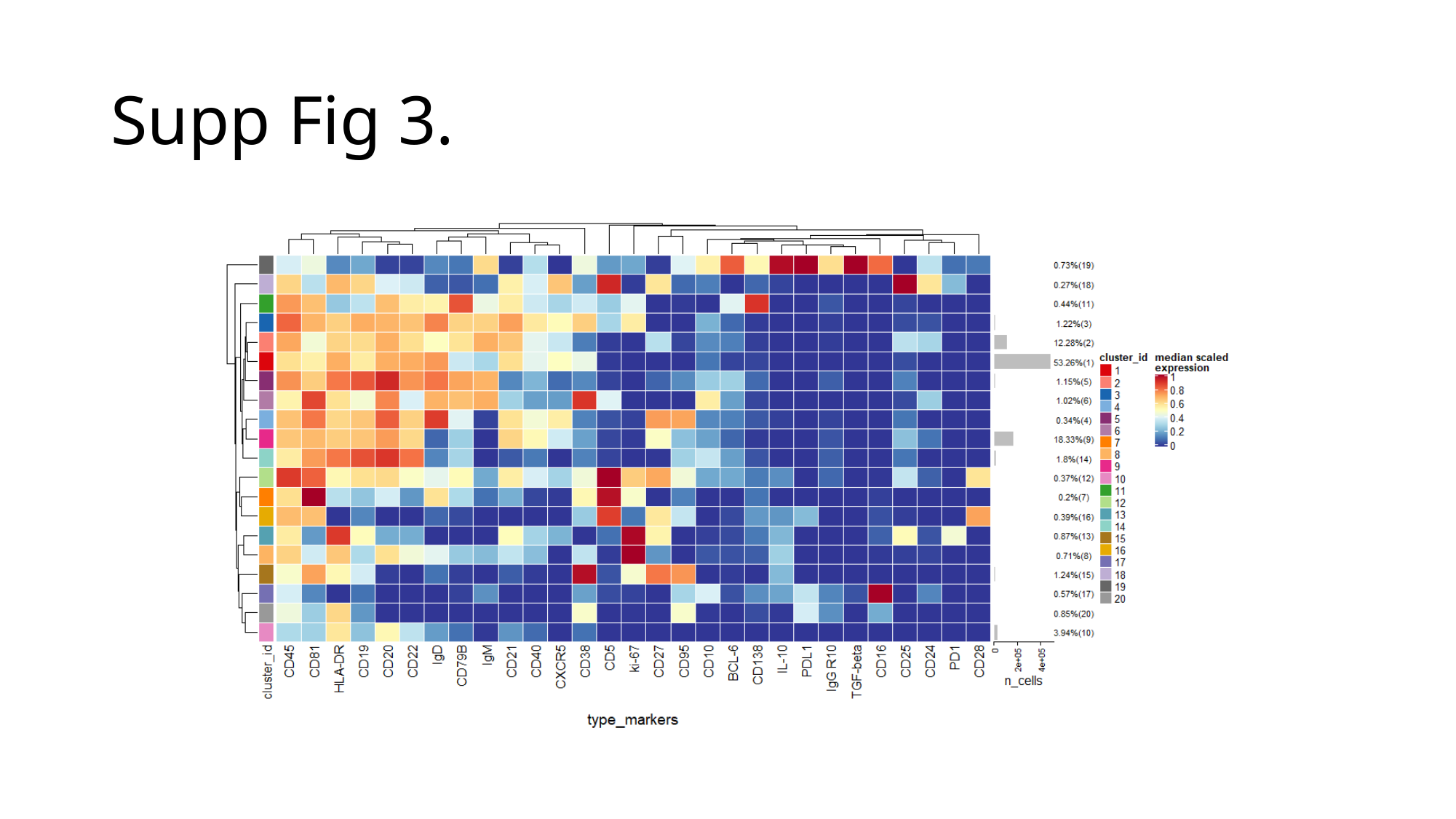

# Supp Fig 3.

## Slide 5
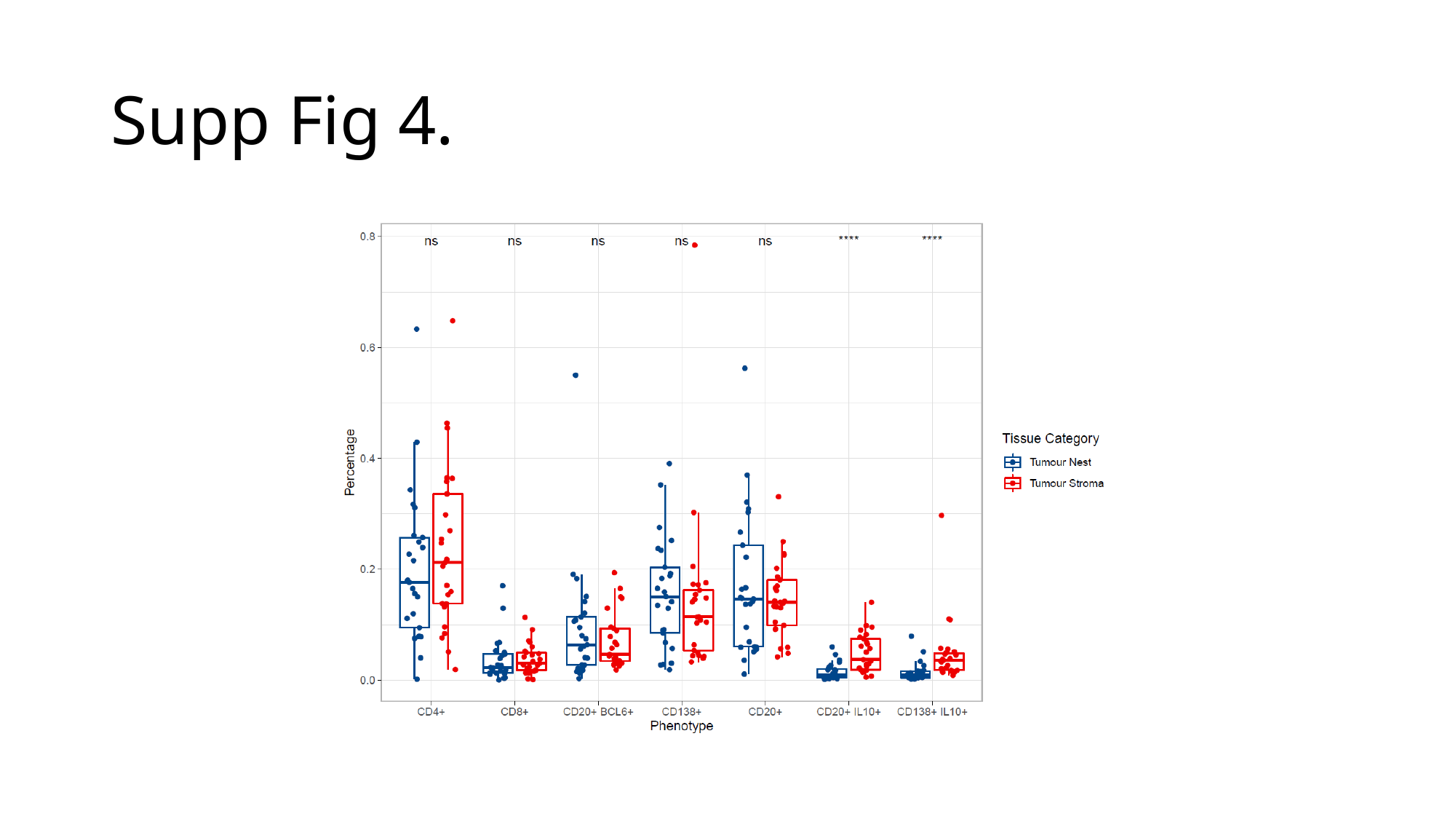

# Supp Fig 4.

## Slide 6
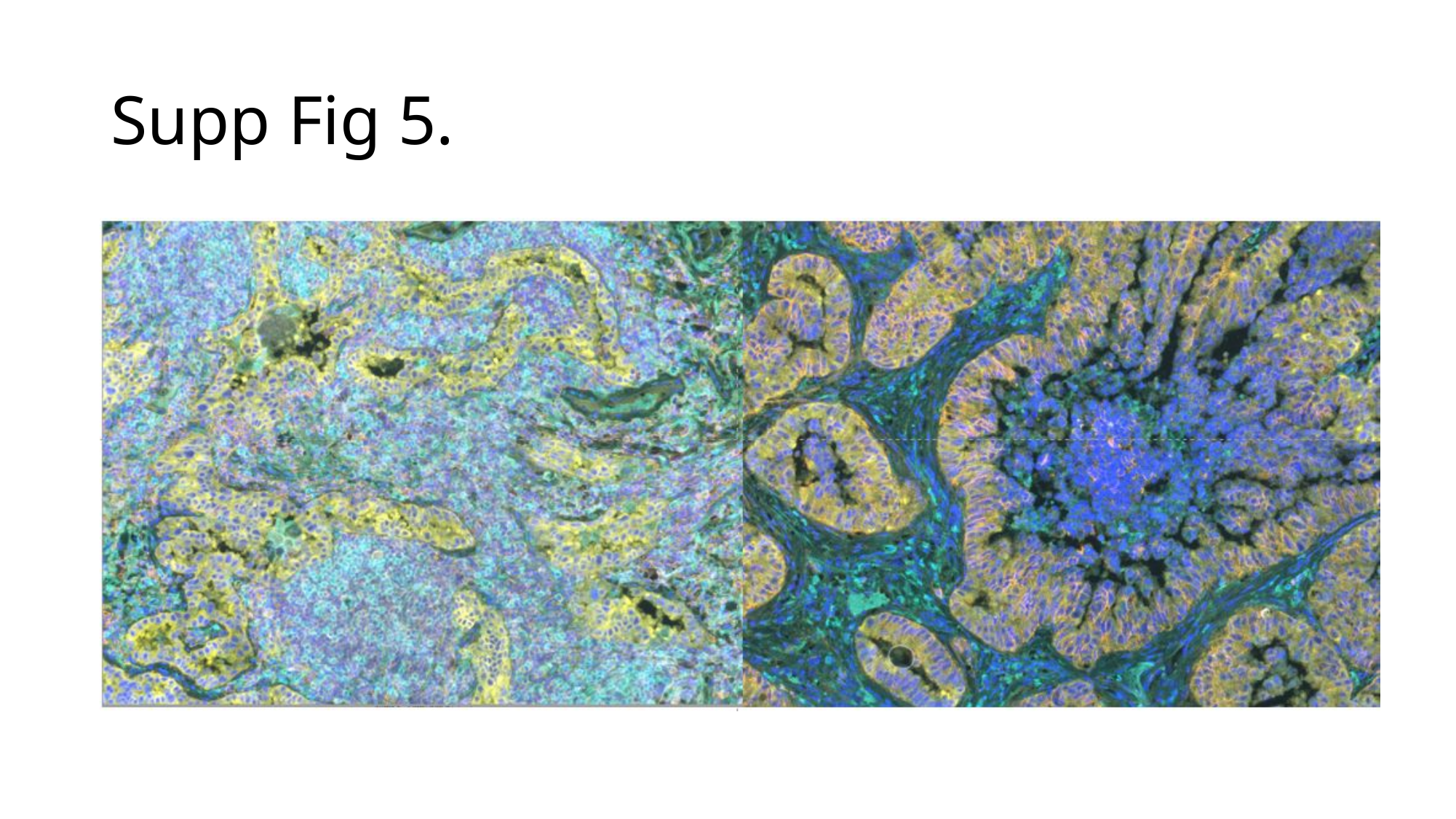

# Supp Fig 5.

## Slide 7
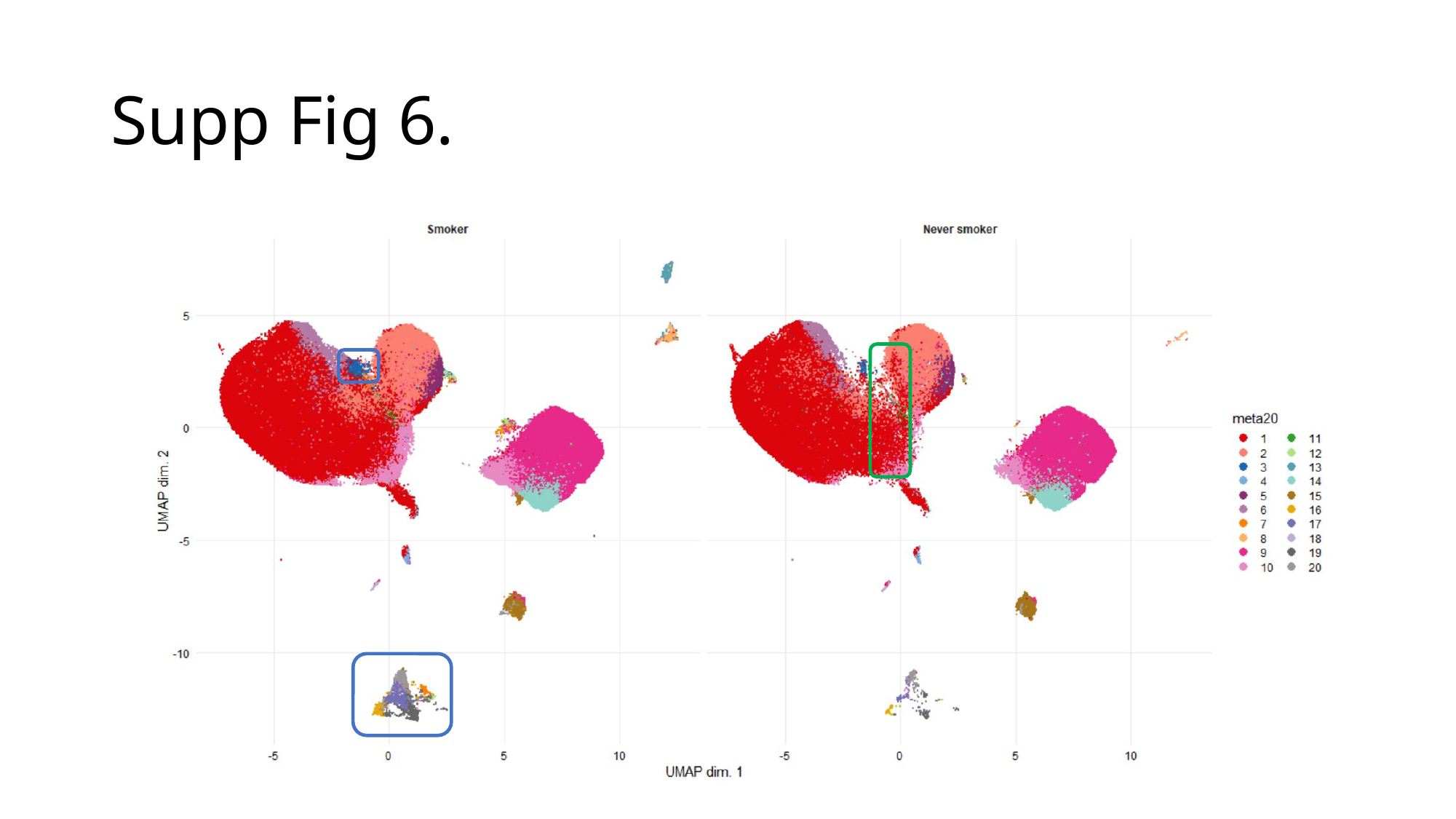

# Supp Fig 6.

## Slide 8
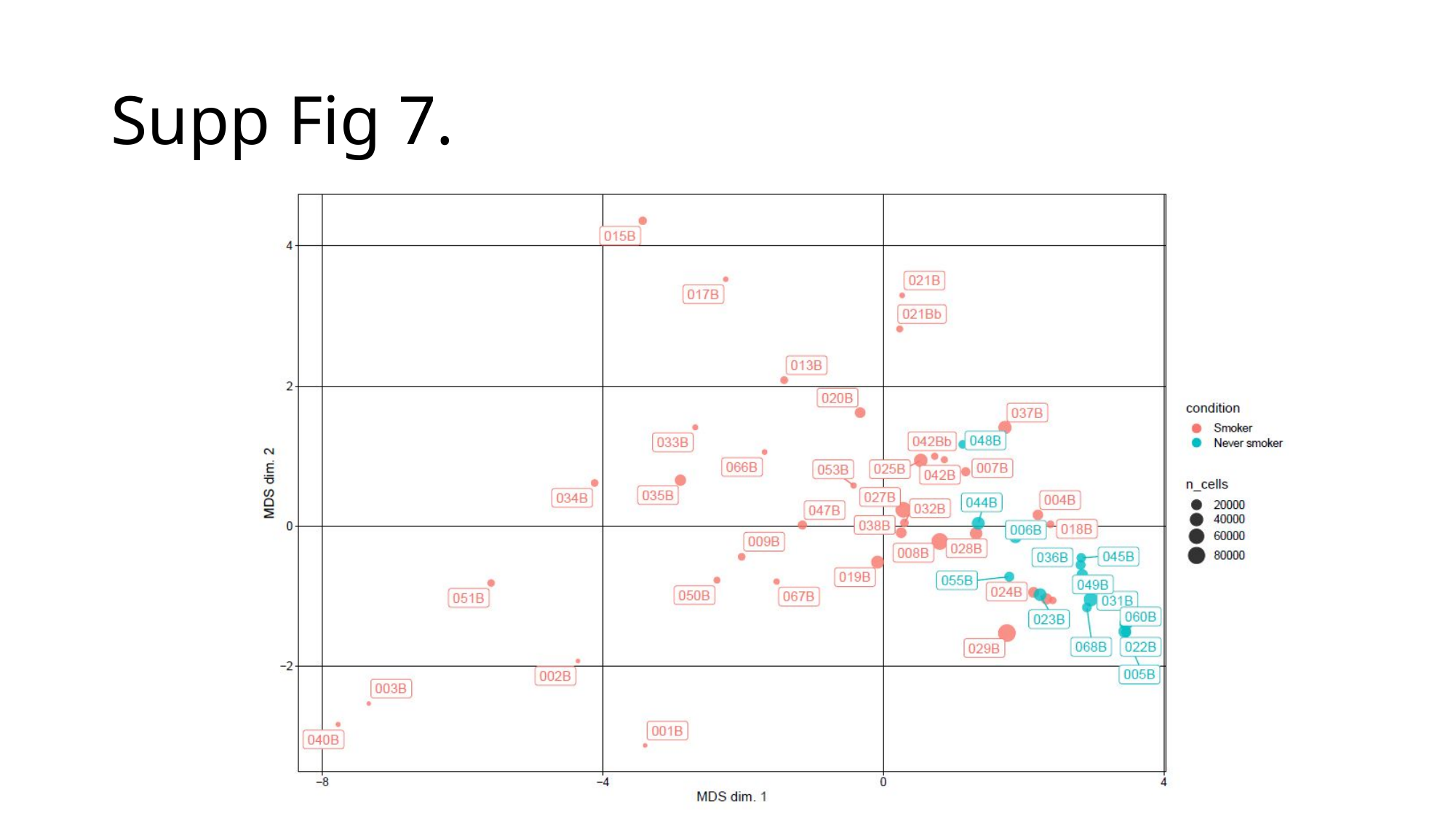

# Supp Fig 7.

## Slide 9
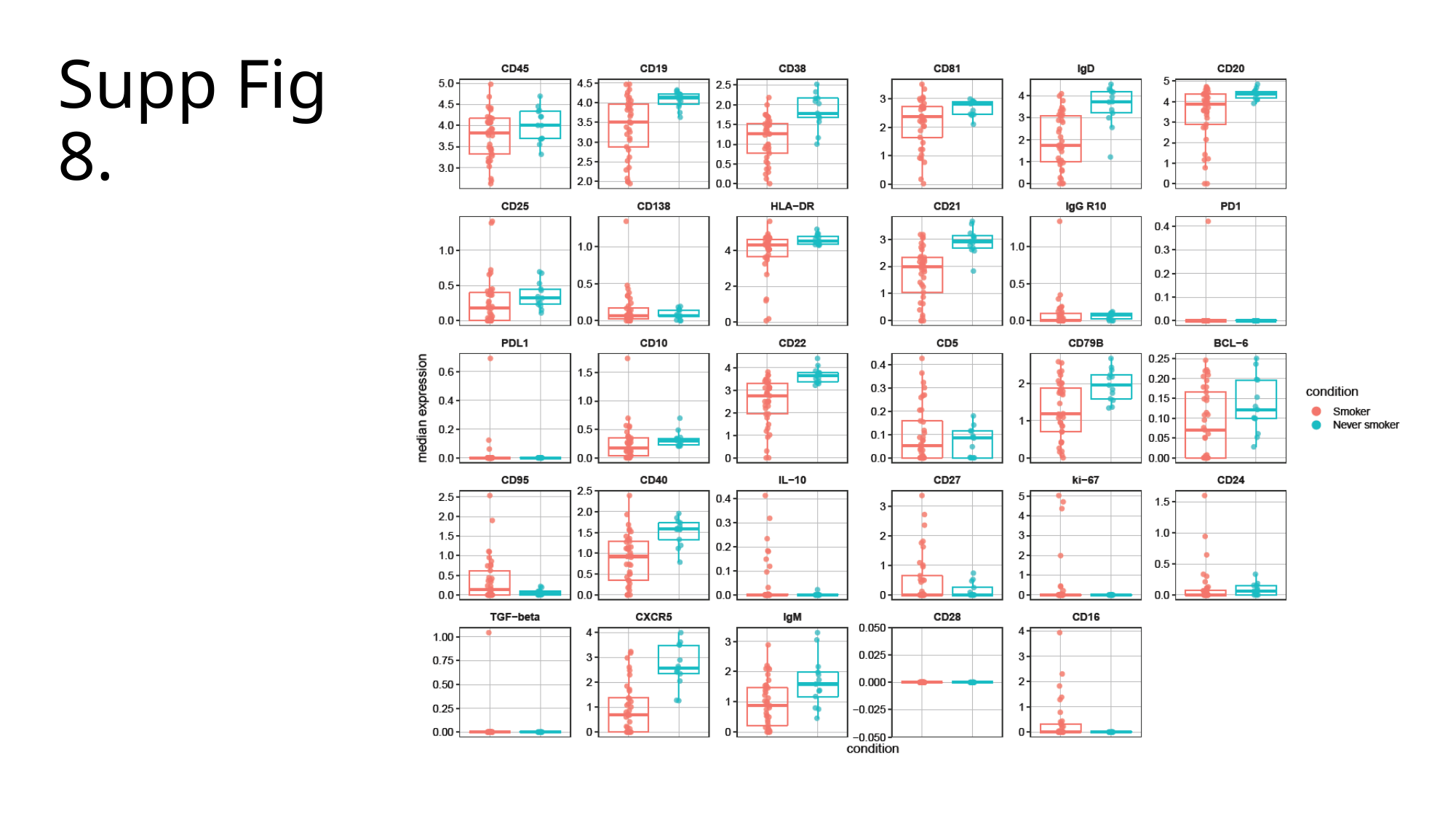

# Supp Fig 8.

## Slide 10
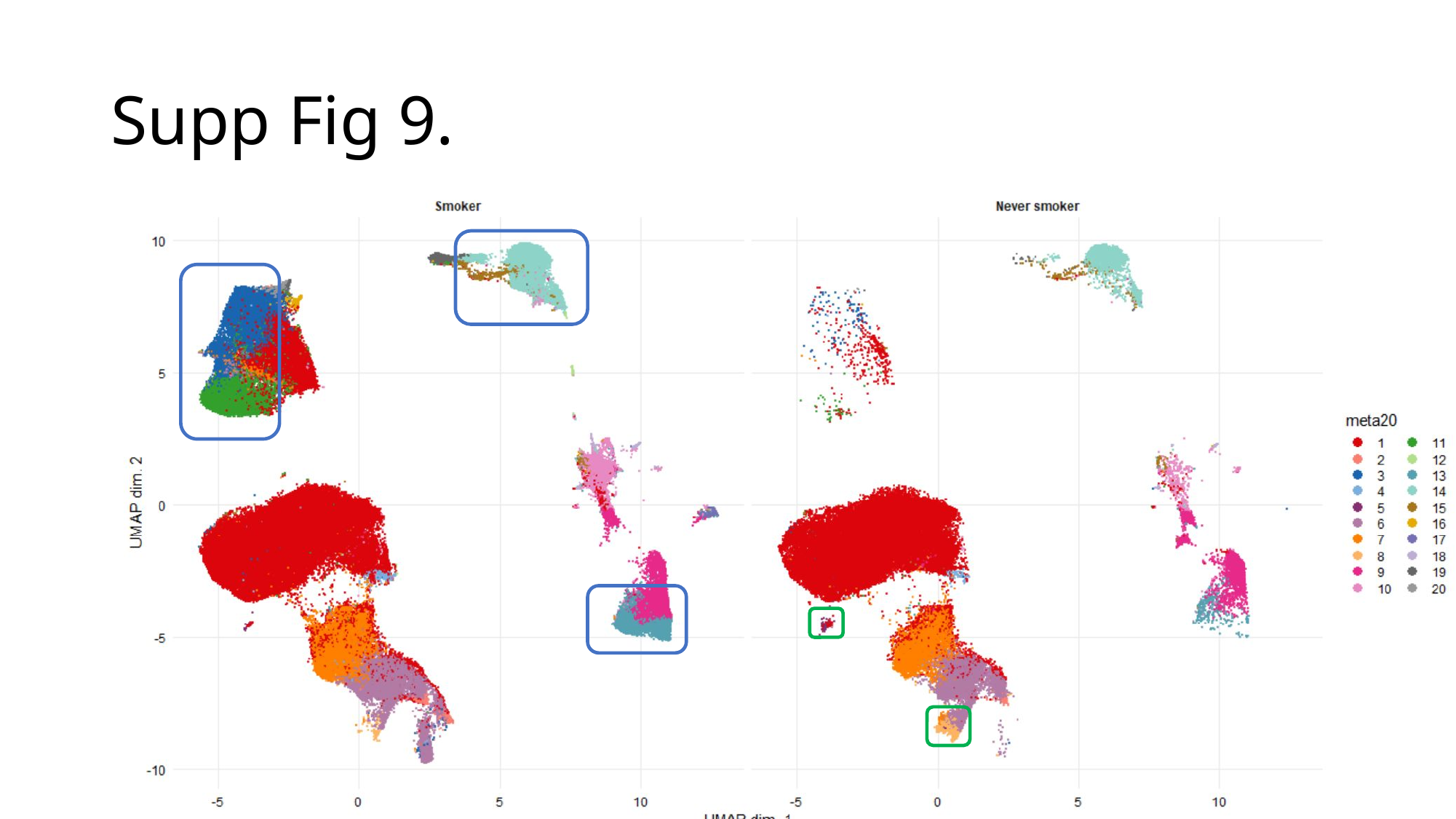

# Supp Fig 9.

## Slide 11
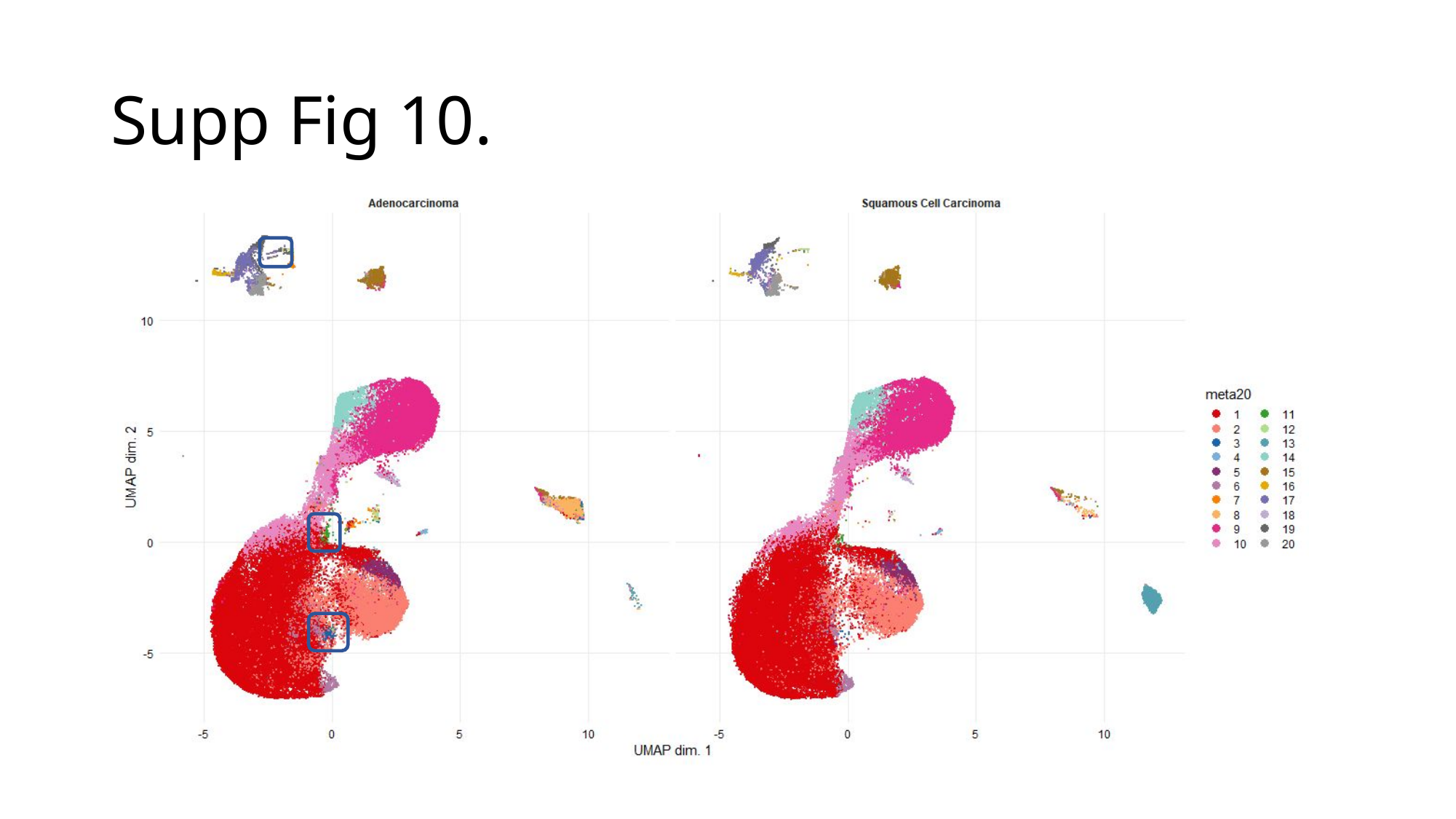

# Supp Fig 10.
